# Dual-mode CRISPRa/i for genome-scale metabolic rewiring in *Escherichia coli*

**DOI:** 10.1093/nar/gkaf818

**Published:** 2025-08-21

**Authors:** Soo Young Moon, Mi Ri Kim, Nan-Yeong An, Myung Hyun Noh, Ju Young Lee

**Affiliations:** Department of Biological Sciences, Korea Advanced Institute of Science and Technology (KAIST), Daejeon 34141, Republic of Korea; Division of Interdisciplinary Bioscience and Bioengineering, Pohang University of Science and Technology (POSTECH), Pohang, Gyeongbuk 37673, Republic of Korea; Research Center for Bio-Based Chemistry, Korea Research Institute of Chemical Technology (KRICT), Ulsan 44429, Republic of Korea; Department of Biological Sciences, Korea Advanced Institute of Science and Technology (KAIST), Daejeon 34141, Republic of Korea; Research Center for Bio-Based Chemistry, Korea Research Institute of Chemical Technology (KRICT), Ulsan 44429, Republic of Korea; Department of Biological Sciences, Korea Advanced Institute of Science and Technology (KAIST), Daejeon 34141, Republic of Korea; Graduate School of Engineering Biology, Korea Advanced Institute of Science and Technology (KAIST), Daejeon 34141, Republic of Korea

## Abstract

CRISPR (clustered regularly interspaced palindromic repeats)-mediated transcriptional regulation is a powerful and programmable approach for controlling gene expression. While CRISPR-based gene repression is well established in bacteria, simultaneous activation and repression remain challenging due to the limited availability of effective bacterial activation domains. Here, we provide an efficient dual-mode CRISPR activation and interference (CRISPRa/i) system that integrates an evolved protospacer adjacent motif (PAM)-flexible dxCas9 with an engineered *Escherichia coli* cAMP receptor protein (CRP). Through systematic optimization of the CRP domains and linkers, we developed a versatile effector capable of precise gene expression control when combined with dxCas9. Our dxCas9–CRP system demonstrated robust activation of upstream regulatory regions and effective repression of coding sequences, enabling targeted and programmable gene regulation. Using dual-fluorescent reporters, we validated the ability of this system to concurrently regulate multiple genes. Furthermore, with pooled guide RNA libraries, we applied the dxCas9–CRP system to increase violacein production in *E. coli* via genome-scale activation and repression in a coordinated manner, successfully identifying key regulatory targets that significantly increase production. Overall, this dual-mode CRISPRa/i system advances the potential for bacterial metabolic pathway rewiring, providing precise and flexible control for a wide range of biotechnological applications.

## Graphical Abstract

**Graphical Abstract:**
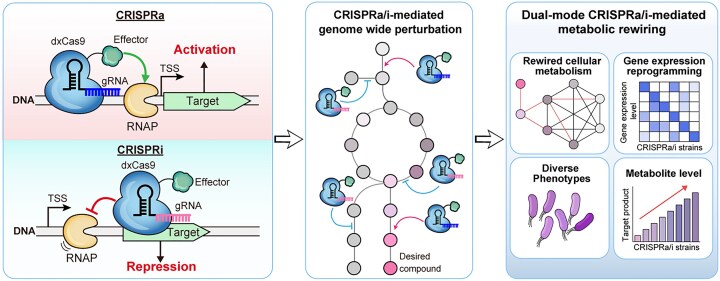

## Introduction

Reprogrammable CRISPR (clustered regularly interspaced palindromic repeats)-mediated gene regulation has emerged as a revolutionary tool in molecular biology, offering unprecedented precision in modulating gene expression across diverse organisms [1, 2]. This technology has enabled the interrogation of gene functions, engineering of cellular systems, and unraveling of complex biological networks with remarkable efficiency. In eukaryotes, CRISPR-based transcriptional control systems have been widely adopted, enabling both the activation of (CRISPRa) and interference with (CRISPRi) target gene expression [3–5]. These tools have facilitated groundbreaking research in areas such as functional genomics [6], drug discovery [7, 8], and cellular reprogramming [9, 10], demonstrating the substantial potential of targeted gene regulation in advancing our understanding of biological systems and developing novel therapeutic strategies.

Despite the notable success of CRISPR-based gene regulation in eukaryotic systems, its application in bacteria faces significant challenges. While CRISPRi has been effectively used for gene repression in bacteria, successful implementations of CRISPRa remain limited [1]. To enable precise, targeted activation (CRISPRa), transcriptional activation domains can be directly linked to a nuclease-dead CRISPR-associated protein 9 (dCas9) that functions solely as a DNA-binding protein [10]. This dCas9 coupled with an activation domain serves as a precision-tailored regulator designed to (i) bind specific DNA sequences guided by a programmable guide RNA (gRNA) and (ii) activate gene expression mediated by an activation domain that interacts with and recruits the transcriptional machinery.

However, due to the inherent differences between prokaryotic and eukaryotic transcriptional mechanisms, identifying and implementing effective activation domains in bacteria is challenging [11–13]. In prokaryotes, transcription factors can either activate or repress transcription through mechanisms that either enhance the interaction of RNA polymerase with the promoter or prevent RNA polymerase from binding. These effects depend primarily on the transcription factor's binding site relative to core promoter elements [14]. Similarly, bacterial CRISPRa/i systems can function as either activators or repressors depending on the binding site of the dCas9 fused to the activation domains.

Several strategies have been developed to enable simultaneous activation and repression using CRISPRa/i systems in bacteria (Supplementary Table S1). These include direct fusions of dCas9 with RNA polymerase-associated proteins such as the ω-subunit (RpoZ) [15], or phage-derived activators such as AsiA, which modulate the interaction between σ^70^ and RNA polymerase [11]. Others have used enhancer-binding proteins (bEBPs) or recruited native transcription factors such as SoxS using scaffold RNAs engineered with MS2 stem–loops to bind MS2 coat protein (MCP)-fused activators [16, 17].

However, most of these CRISPRa systems have been limited to the activation of specific gene targets on plasmids or at defined genomic loci. They lack robust, scalable frameworks for genome-scale activation and repression—an essential requirement for comprehensive gene network mapping and advanced metabolic engineering. This limitation has constrained our ability to conduct multiplex genomic perturbation studies and comprehensively manipulate bacterial cellular processes, hindering the full potential of CRISPRa/i-based gene regulation in these systems. To address these limitations, a broader repertoire of bacterial effector domains is needed—ideally within a compact, single-effector framework capable of both activation and repression without interfering with essential cellular functions. Furthermore, most prior studies demonstrated CRISPRa functionality exclusively in *Escherichia coli*, and its application to other bacterial species—particularly through simultaneous implementation of the same CRISPRa system—remains limited.

**Figure 1.**
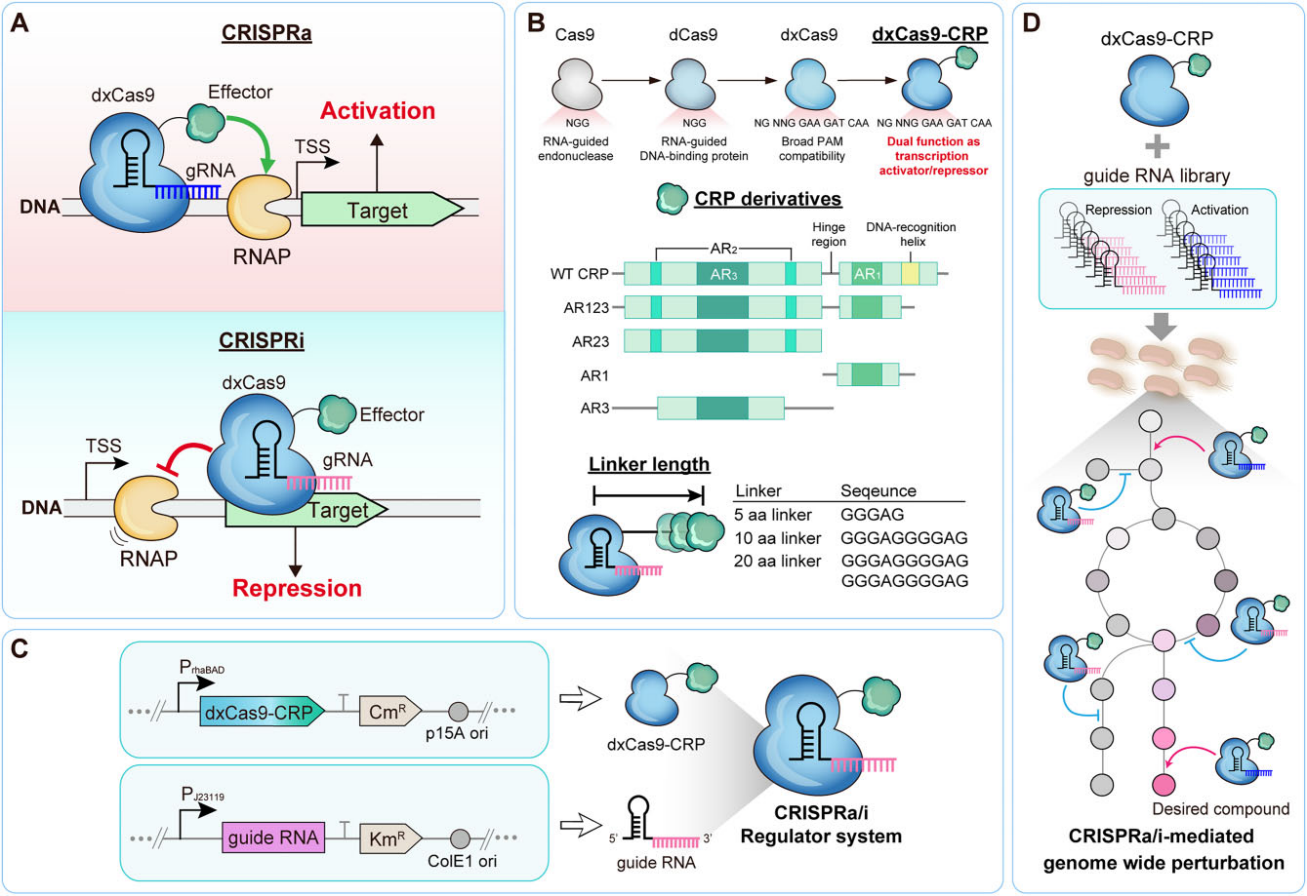
Design and construction of the dual-mode CRISPRa/i system in *E. coli*. (**A**) Schematic of dual-mode CRISPRa/i system-mediated gene regulation. The bacterial CRISPRa/i system uses dxCas9 combined with a transcriptional effector domain and a targeting gRNA. The dxCas9 combined with an effector domain (dxCas9–effector complex) can both activate and repress a target gene depending on the targeting locus guided by gRNA. For activation (CRISPRa), the dxCas9–effector complex binds upstream of the promoter, where the effector domain recruits RNA polymerase to initiate transcription. For repression (CRISPRi), the dxCas9–effector complex binds near or within the coding region to physically block RNA polymerase progression. (**B**) Architecture of the dual-mode CRISPRa/i system. The system consists of an evolved, PAM-flexible dxCas9 and an *E. coli* CRP effector domain, connected with a linker (referred to as dxCas9–CRP). For effective regulation driven by an effector domain, several CRP derivatives were designed with different combinations of activating regions: AR123 (all three regions), AR23, AR1, and AR3, as well as the wild-type CRP as a control. These variants were combined with dxCas9 using flexible linkers of varying lengths (5, 10, or 20 amino acids). (**C**) The dxCas9–CRP was driven by a rhamnose-inducible promoter (P_rhaBAD_), and gRNA expression was placed under the control of a strong, constitutive promoter (P_J23119_). (**D**) The CRISPRa/i system with genome-wide dual-gRNA libraries can create genome-scale perturbation libraries, offering a versatile tool for wide-ranging applications in bacterial engineering and biotechnology.

In this study, we present a novel approach to address these challenges in bacterial systems, specifically in *E. coli*. Our strategy employs the well-characterized global transcriptional regulator cAMP receptor protein (CRP), a versatile protein known to regulate >490 genes involved in carbon metabolism, nutrient uptake, and energy homeostasis in *E. coli* [18, 19]. By engineering CRP variants and linking them with an evolved, protospacer adjacent motif (PAM)-flexible dCas9 variant (dxCas9) [5, 20], we have developed an improved dual-mode CRISPRa/i system (Fig. 1). This system is designed to mediate simultaneous activation and repression across both plasmid and genomic targets, offering a versatile tool for precise gene regulation in bacteria. Our approach not only expands the targeting range of the CRISPR system through the use of dxCas9 but also provides a single effector capable of both gene activation and repression in a scalable and cross-species-compatible manner. We demonstrate the efficacy of our system by successfully applying it to enhance the production of the valuable compound, violacein, in *E. coli*. Finally, we extended our system's applicability to another bacterial species, *Pseudomonas putida*, demonstrating its potential as a broadly applicable platform for bacterial transcriptional reprogramming. These results highlight the robustness and versatility of our CRISPRa/i system, suggesting its potential for wide-ranging applications in bacterial engineering and biotechnology.

## Materials and methods

### Plasmid and strain construction

All plasmids used in this study were constructed using the NEBuilder® HiFi DNA Assembly Kit (New England Biolabs, Ipswich, MA, USA). *E. coli* DH5α was used for cloning, and *E. coli* MG1655 was employed for all experiments involving CRISPRa/i systems. The bacterial strains, plasmids, and primers used in this study are listed in Supplementary Tables S2 and S3.

The CRISPRa/i system components were assembled as follows: the dxCas9(3.7) gene from *Streptococcus pyogenes* was fused to *E. coli* CRP derivatives via a flexible linker and placed under the control of the rhamnose-inducible P_rhaBAD_ promoter (Supplementary Table S4) [20]. The pACCRi vector, used as the backbone for the CRISPRa/i plasmid, was a gift from Lee *et al.* [21]. All gRNAs, including a non-targeting gRNA (an off-target control) with no predicted matches (≥12 bp adjacent to an NGG PAM) in the *E. coli* MG1655 genome, and the reporter plasmids used in this study are listed in Supplementary Tables S5 and S6. gRNAs were expressed from the constitutive BBa_J23119 promoter on a separate plasmid, psgRNA (Supplementary Table S2). For the reporter plasmids, the green fluorescent protein (GFP) and mCherry genes were expressed from the weak BBa_J23117 promoter (for CRISPRa) and the strong BBa_J23119 promoter (for CRISPRi), respectively. All promoters used in this study were obtained from the iGEM Registry of Standard Biological Parts (http://parts.igem.org). For violacein production, the plasmid pETM6-vioABECD (catalog #66 535) was used (Addgene, Watertown, MA, USA).

### Culture conditions

*E. coli* strains harboring CRISPRa/i, gRNA, and reporter plasmids were cultured in Luria–Bertani (LB) medium supplemented with the appropriate antibiotics: ampicillin (100 μg/ml), kanamycin (50 μg/ml), and chloramphenicol (25 μg/ml). The cultures were incubated at 37°C with shaking at 200 rpm unless otherwise specified. For induction of dxCas9 protein expression, 1 mM l-rhamnose was added to the culture medium when the optical density at 600 nm (OD_600_) reached 0.4–0.6.

### Design and construction of the gRNA library

The genome sequence of NC_000913.3 was used to design the activation gRNA library for *E. coli* MG1655. A custom Python script utilizing the Biopython library was developed to design gRNAs targeting both the leading and lagging strands of each gene, considering both the NGG and NG PAM sequences to expand the targeting range of our CRISPRa/i system. For each gene, the script scanned the region between base pairs 190 and 250 upstream of the start codon (ATG) for potential PAM sites. The script identified the 5′-NGG-3′ and 5′-NG-3′ sequences on the non-template strand, which corresponded to 3′-NCC-5′ and 3′-NC-5′ on the template strand, respectively.

For each identified PAM, a 20 nt sequence immediately upstream (5′ direction) was extracted. When multiple gRNAs were designed for the same gene, manual editing was performed using the following criteria: (i) NGG PAM preference over NG, GAA, or GAT sites for optimal efficiency [20]; (ii) elimination of redundant targeting within operons; and (iii) selection of one optimal gRNA per gene to maximize library coverage. This step increased the library efficiency and minimized redundancy. The final activation gRNA library was designed to target 3,640 genes, covering all genes in the *E. coli* genome for potential regulation via our CRISPRa/i system. The designed gRNAs were synthesized as oligonucleotides (Twist Bioscience, San Francisco, CA, USA) and cloned into the gRNA expression vector using the Golden Gate Assembly method [22]. The resulting gRNA plasmid library was transformed into *E. coli*, creating a pool of strains, each expressing a unique gRNA targeting a specific gene.

For gene repression, we utilized a previously established genome-wide inhibition library (EcoWG1, catalog #131 625, Addgene), which includes an average of five gRNAs per gene, totaling 21,417 gRNAs. This pre-existing library was incorporated into our study to complement our custom-designed activation library, providing a comprehensive library for both gene activation and repression in our CRISPRa/i system.

### Fluorescence measurement

*E. coli* cells were inoculated in a 50 ml bioreactor containing 10 ml of LB supplemented with the appropriate antibiotics and 1 mM l-rhamnose, and grown at 37 °C with shaking at 200 rpm for 24 h. The *E. coli* culture was centrifuged at 13,000 rpm for 5 min to harvest the cells. The cell pellet was washed with phosphate-buffered saline (PBS) and resuspended to a final OD_600_ of 1. Fluorescence was measured using a SpectraMax Gemini XPS plate reader (Molecular Devices, San Jose, CA, USA) in clear-bottomed black 96-well plates. For GFP detection, the excitation/emission wavelengths were set to 488/509 nm, and for mCherry detection to 587/610 nm.

### Violacein production, extraction, and quantification

For violacein production, *E. coli* cells were cultivated in a 50 ml bioreactor containing 10 ml of LB medium with 1 mM l-rhamnose and 0.01 mM isopropyl-β-d-thiogalactopyranoside (IPTG) at 200 rpm and 37°C for 24 h. For violacein extraction, *E. coli* cells equivalent to 4 OD_600_ were centrifuged at 13,000 rpm for 5 min to obtain a cell pellet. The harvested cells were resuspended in 600 μl of methanol solution with lysing matrix C. The mixture was then mechanically disrupted using a FastPrep-24 5G homogenizer (MP Biomedicals, Irvine, CA, USA). After filtration using 0.2 μm syringe filters, the violacein extracted from the methanol solution was analyzed using an Agilent 1260 Infinity high-performance liquid chromatography (HPLC) system equipped with a UV detector at 570 nm (Agilent Technologies, Santa Clara, CA, USA). The samples were separated on a ZORBAX Eclipse XDB-C18 column (Agilent Technologies) at 30°C using a stepwise gradient elution at a flow rate of 1.0 ml/min for 15 min: 0–5 min, 100% to 20% solvent A (H_2_O with 0.1% acetic acid); 5–8 min, 20% solvent A; 8–12 min, 20% to 100% solvent A; 12–15 min, 100% solvent A [23]. The violacein concentrations were determined using standards prepared with commercially available violacein (Sigma-Aldrich, St. Louis, MO, USA).

## Results

### Design and optimization of the dual-mode CRISPRa/i system in *E. coli*

To develop an improved dual-mode CRISPRa/i system in *E. coli*, we combined dxCas9 with *E. coli* CRP via a previously described flexible 10 amino acid linker (Fig. 1A, B) [20]. The dxCas9 component provides expanded targeting capabilities due to its ability to recognize a broader range of PAM sequences, including NG, GAA, and GAT, in addition to the canonical NGG sequence [5]. CRP, a well-characterized global transcriptional regulator in *E. coli*, was selected as the effector domain due to its ability to activate a broad set of genes driven by σ^70^-dependent promoters, which are responsible for transcribing the majority of genes in *E. coli* (Supplementary Note 1; Supplementary Fig. S4) [1, 19, 24]. CRP consists of three activating regions: AR1 and AR2 at the N-terminus and AR3 along with a DNA-binding motif at the C-terminus (Fig. 1B). These activating regions promote transcription by recruiting RNA polymerase to target promoters through their interaction with the α-subunit of RNA polymerase—mediated by AR1 and AR2—and with the σ^70^ factor via AR3.

We designed several CRP derivatives to optimize the CRISPRa/i system on the basis of its structural information [24]. Given that different activating regions might uniquely contribute to gene regulation in the context of our CRISPR system, these CRP derivatives included variants containing different combinations of the activating regions: CRP_AR123_ (all three regions), CRP_AR23_, CRP_AR1_, and CRP_AR3_, as well as the wild-type CRP as a control (Fig. 1B; Supplementary Table S4). Each of these CRP variants was linked to dxCas9, generating a series of dxCas9–CRPs to systematically evaluate the contribution of each activating region to the overall performance of our CRISPRa/i system. In addition, expression of dxCas9 and dxCas9–CRP derivatives did not affect cell growth in the absence of gRNA, indicating minimal physiological burden on the *E. coli* strain (Supplementary Fig. S2). The CRISPRa/i components, including dxCas9–CRPs and gRNA, were assembled on two separate plasmids (Fig. 1C). The dxCas9–CRPs were placed under the control of the rhamnose-inducible P_rhaBAD_ promoter, while gRNAs were expressed from the constitutive BBa_J23119 promoter.

**Figure 2.**
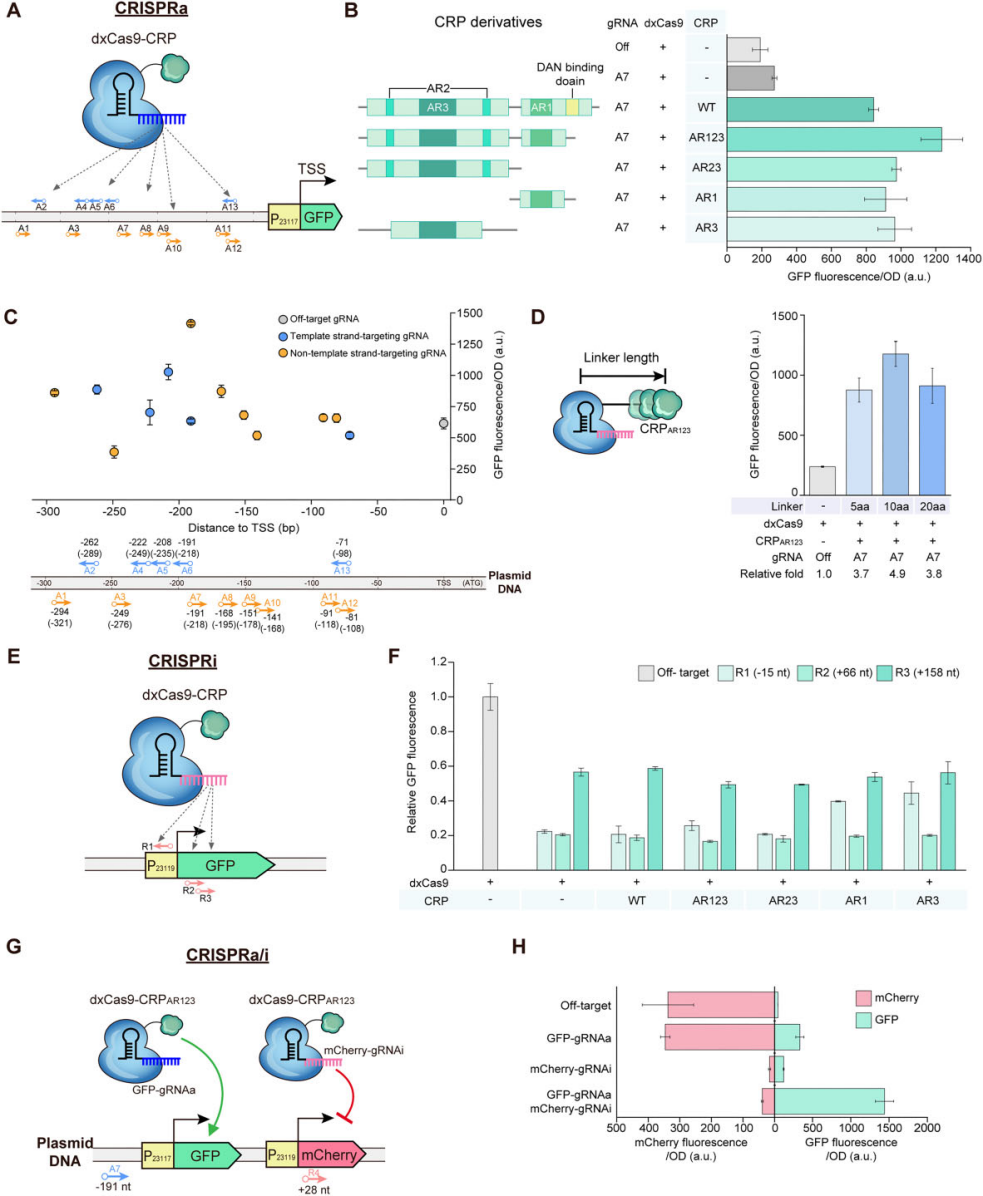
CRISPRa/i mediates multiplexed gene activation and repression. (**A**) Reporter system for measuring CRISPRa activity. Thirteen gRNA target sites were designed and inserted between base pairs –294 and –70 upstream of the TSS of the GFP reporter gene on a reporter plasmid. (**B**) Engineering of *E. coli* CRP derivatives to improve the CRISPRa/i capability. The CRP derivatives included variants with different combinations of activating regions: AR123, AR23, AR1, and AR3, along with the wild-type CRP as a control. Each CRP variant was linked to dxCas9 via a flexible 10 amino acid linker. (**C**) Screening optimal gRNA-binding sites. Different gRNAs were paired with dxCas9–CRP_AR123_ to identify the most effective gRNA-binding site. The gRNA targeting the A7 site showed the most efficient activation. Distances associated with each gRNA indicate their position relative to the TSS, with values in parentheses indicating distance from the start codon (ATG). Blue arrows indicate gRNAs targeting the template strand, and orange arrows indicate those targeting the non-template strand. The off-target gRNA (gray) was used as a negative control. (**D**) Optimization of the spatial relationship between dxCas9 and the CRP_AR123_ domain. Three linker lengths (5, 10, and 20 amino acids) between dxCas9 and CRP_AR123_ were tested, with the 10 amino acid linker exhibiting the highest activation efficiency. (**E**) Reporter system for measuring CRISPRi activity. Three gRNAs were designed to bind to different regions around the promoter (J23119) or the GFP reporter-coding region. (**F**) CRISPRi driven by dxCas9 combined with CRP variants. CRISPRi effectively blocked both transcriptional initiation and elongation. dxCas9–CRP_AR123_ showed particularly strong repression at the R2 site. (**G**) Reporter system containing the GFP and mCherry genes for simultaneously measuring CRISPRa/i activity. The optimized dxCas9–CRP_AR123_ system can mediate CRISPRa for GFP and CRISPRi for mCherry. (**H**) Simultaneous activation and repression of multiple genes using the CRISPRa/i system. The graph shows the relative fluorescence intensities of GFP and mCherry, driven by both gRNAs targeting GFP for activation and mCherry for repression. When both gRNAs were applied simultaneously, we observed concurrent GFP activation and mCherry repression. All data shown are from at least three biological replicates, and the error bars indicate the SD.

To evaluate and optimize the gene activation capability of our CRISPRa/i system, we utilized a GFP reporter gene under the control of a weak constitutive promoter (BBa_J23117). We included a 170 bp long PAM-rich region upstream of the promoter to ensure ample targeting opportunities for our system (Fig. 2A). Thirteen different gRNAs (A1–A13) targeting positions from base pairs –294 to –70 relative to the transcription start site (TSS) were designed to identify the optimal binding site for activation (Fig. 2A–C; Supplementary Table S5; Supplementary Fig. S1). The TSS corresponds to the first nucleotide of the ribosome-binding site (RBS) immediately downstream of the promoter [17]. All CRP variants lacking the DNA-binding domain (CRP_AR123_, CRP_AR23_, CRP_AR1_, and CRP_AR3_), as well as the wild-type CRP, showed similar levels of activation (Fig. 2B). Each variant contributed to a >4-fold increase in GFP expression compared with the off-target control, indicating that the native DNA-binding function of CRP does not significantly interfere with dxCas9-mediated targeting.

Given that AR1 and AR2 interact with the RNA polymerase α-subunit, while AR3 interacts with the σ^70^ subunit, we hypothesized that the CRP_AR123_ variant with all three activating regions could offer more diverse and robust interactions with the transcriptional machinery under variable cellular conditions [24]. Although similar activation levels were observed for all CRP variants (Fig. 2B), we focused on CRP_AR123_ for further optimization of our CRISPRa/i system, anticipating that it might confer advantages in more complex regulatory scenarios.

Using dxCas9–CRR_AR123_, we conducted a comprehensive screening of gRNA target sites. Among the 13 different gRNAs tested, we found that targeting the region 191 bp upstream of the TSS (designated as A7) resulted in the strongest activation, with a 2.3-fold increase in GFP fluorescence relative to an off-target control (Fig. 2C). This optimal binding site may provide ideal positioning for the dxCas9–CRP_AR123_ complex to effectively recruit and interact with the transcriptional machinery. We also explored the impact of the spatial relationship between dxCas9 and CRP_AR123_ by testing different linker lengths. We connected dxCas9 and CRP_AR123_ with 5, 10, and 20 amino acid linkers, hypothesizing that the linker length might affect the flexibility and positioning of the CRP domain relative to the DNA-bound dxCas9 [17]. The 10 amino acid linker exhibited the highest level of activation, resulting in a 4.9-fold increase in GFP fluorescence (Fig. 2D).

### CRISPRa/i-mediated effective dual-mode regulation

In parallel, to assess the gene repression capabilities of our dxCas9–CRP_AR123_ system, we utilized a GFP reporter gene under the control of a strong constitutive promoter (BBa_J23119). We designed three gRNAs targeting different positions: R1 (–15 bp from the TSS), R2 (+66 bp), and R3 (+158 bp) (Fig. 2E; Supplementary Table S5; Supplementary Fig. S1). These targets were chosen to evaluate their ability to interfere with both transcriptional initiation and elongation, as the dxCas9–gRNA complex competes with RNA polymerase for binding to the promoter and open reading frame (ORF) of the target gene.

We found that the dxCas9–CRP_AR123_ system effectively repressed GFP expression at all three different target sites, indicating its ability to interfere with both transcriptional initiation and elongation (Fig. 2F). dxCas9–CRP_AR123_ showed strong repression when targeting the promoter region (R1) and the early coding sequence (R2), with R2 exhibiting the most potent effect, resulting in an 83% decrease in GFP expression compared with the off-targeting gRNA control. However, targeting further downstream in the coding sequence (R3) showed relatively lower repression efficiency. This reduced effect at R3 might be due to the ability of some RNA polymerase molecules to overcome the roadblock or the potential for transcription reinitiation downstream of the binding site [25]. Notably, the repression efficiency of our dxCas9–CRP_AR123_ system was comparable with that of dxCas9 without CRP, indicating that linking with CRP does not negatively impact the repressive capabilities of dxCas9.

To further determine whether our CRISPRa/i system enables simultaneous activation and repression of multiple genes, we constructed a reporter plasmid encoding both the GFP and mCherry genes. The GFP gene was placed under the control of a weak promoter (BBa_J23117), while mCherry was driven by a strong promoter (BBa_J23119) (Fig. 2G). Using the optimized dxCas9–CRP_AR123_ system, we targeted GFP for activation (A7, GFP–gRNAa targeting at –191 bp) and mCherry for repression (R4, mCherry–gRNAi targeting at +28 bp), respectively (Supplementary Table S5; Supplementary Fig. S1).

We observed a remarkable 8.6-fold increase in GFP expression concurrent with a 9-fold decrease in mCherry expression when both gRNAs (GFP–gRNAa and mCherry–gRNAi) were expressed (Fig. 2H). This result demonstrates the ability of our CRISPRa/i system to simultaneously activate and repress different genes. Interestingly, we observed that the activation of GFP was significantly greater when mCherry was repressed than when only the GFP-activating gRNA was expressed. We speculated that this increased activation in the presence of both gRNAs might be due to the redistribution of cellular resources [26, 27]. Specifically, through repression of strongly expressed mCherry, more cellular resources might become available for GFP expression. This hypothesis is supported by previous modeling and experimental studies showing that highly expressed heterologous pathways or genetic modules can impose a measurable translational load by sequestering ribosomes and other limited cellular resources, thereby influencing the expression dynamics of co-expressed genes (Fig. 2H) [23, 24].

### CRISPRa/i-mediated multiplex pathway optimization at the genome scale

An important practical application of the CRISPRa/i system is to interrogate complex and multiplexed genomic perturbations to control metabolic pathways. Activating key pathway enzymes while repressing competing enzymes is a common approach to optimize metabolic flux toward producing target products, leading to significant improvements in product yields. As a proof of concept, beyond the reporter genes in plasmids (Fig. 2), we explored the dual-mode regulatory capabilities of our CRISPRa/i system by targeting endogenous *E. coli* genes to redirect metabolic flux for improving the production of target metabolites.

**Figure 3.**
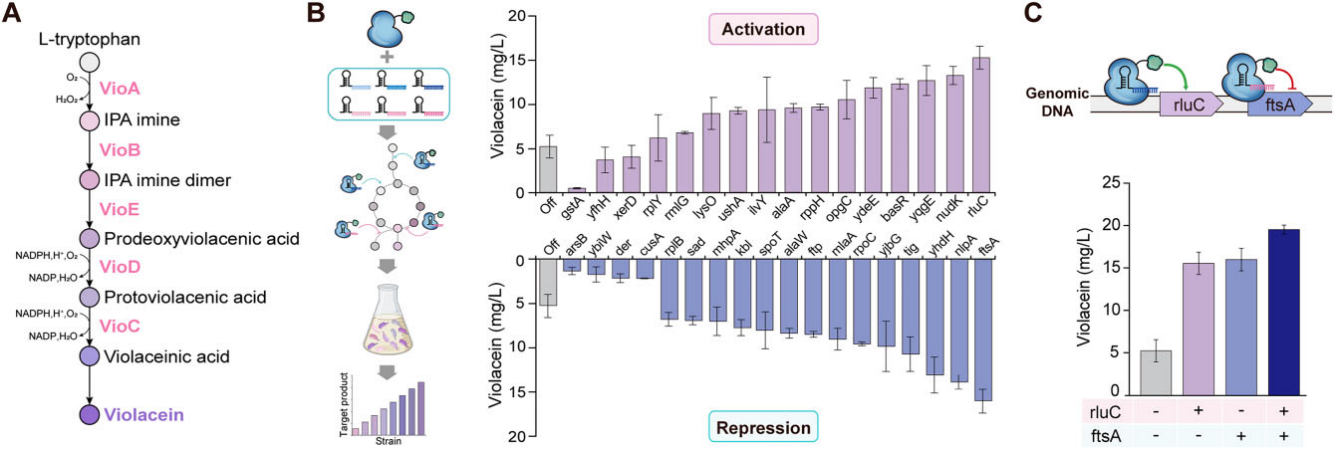
CRISPRa/i-mediated genome-wide screening for increased violacein production. (**A** and **B**) Schematic of the genome-wide screening strategy for CRISPRa/i-mediated violacein production. A pooled gRNA library targeting all genes in the *E. coli* genome was introduced into the violacein-producing *E. coli* strain. Colonies exhibiting varying purple intensities were selected and analyzed for violacein production. The selected colonies possessed distinct gRNAs corresponding to specific genetic perturbations. (**C**) Synergistic enhancement of violacein production through simultaneous activation of *rluC* and repression of *ftsA*, identified as the two most effective genes from the gRNA screening. All data shown are from at least three biological replicates, and the error bars indicate the SD.

We selected the biosynthetic pathway producing violacein, a purple-colored antitumor compound widely used in the pharmaceutical, nutraceutical, and cosmetic fields. For the production of violacein in *E. coli* (Fig. 3A), we used the pVio plasmid containing a five-gene pathway: *vioA*, *vioB*, *vioC*, *vioD*, and *vioE*, originating from *Chromobacterium violaceum* [23]. A previous study demonstrated the application of CRISPRa/i to enhance violacene production by directly modulating the expression of three genes (*vioA*, *vioD*, and *vioC*) [5]. In contrast, our study did not directly target the violacein biosynthetic genes; instead, we examined whether our CRISPRa/i system could optimize metabolic fluxes toward enhanced violacein synthesis by modulating the expression of endogenous *E. coli* genes at the genome scale. The violacein-producing *E. coli* cells containing pVio were transformed with a pooled library of 25,057 individual gRNAs (3,640 activating gRNAs and 21,417 repressing gRNAs), with each transformed cell receiving a single gRNA for either activation or repression (Fig. 3B). We observed that the transformants from the gRNA library exhibited colonies with varying intensities of purple color, which directly reflected the individual differences in violacein production. We picked colonies exhibiting varying purple intensities and observed that the total amount of violacein produced varied substantially among the different colonies. The selected colonies possessed distinct gRNAs corresponding to each perturbation (Fig. 3B; Supplementary Table S6).

As shown in Table 1, we identified multiple gRNAs that significantly affected violacein production compared with the off-target gRNA control (Supplementary Table S7). The gRNA targeting *rluC* exhibited the most significant effect among the activation targets, increasing violacein production by ∼2.9-fold (Fig. 3B; Supplementary Fig. S3). RluC, which encodes a pseudouridine synthetase, catalyzes the site-specific modification of 23S rRNA, essential for ribosome assembly and stability. The activation of *rluC* presumably enhances translational capacity by promoting ribosome function, hence inducing more efficient protein synthesis. This enhancement of the cellular translation machinery appears to particularly benefit the expression of violacein biosynthetic pathway genes [28, 29]. From the repression library screening, the most effective target was *ftsA*, whose repression led to a 3.0-fold increase in violacein production. FtsA is an essential cell division protein that works in concert with FtsZ to form the bacterial division septum [30]. The significant improvement in violacein production upon *ftsA* repression can be attributed to two potential mechanisms. First, the inhibition of cell division leads to cell elongation, resulting in increased cellular volume and potentially higher accumulation of violacein per cell [31]. Second, the reduction in division-related processes may redirect cellular resources toward secondary metabolism, consistent with studies showing enhanced metabolite production in growth-modulated bacterial cells [23, 32, 33]. Together, these combined effects of enhanced cellular capacity through elongation and reallocation of resources probably contribute to the increased production of violacein.

**Table 1.** Identification of genes influencing violacein production in *E. coli* through the CRISPRa/i system

gRNA	Targeted gene	Function	Violacein (mg/l)
**Activation**	Off-target	–	5.37 (±1.33)
	*opgC*	Molybdate transport system ATP-binding protein	10.88 (±2.26)
	*ydeE*	Putative transcriptional regulator	12.27 (±1.20)
	*basR*	OmpR family, response regulator BasR	12.72 (±0.60)
	*yqgE*	MFS transporter, YQGE family, putative transporter	13.12 (±1.76)
	*nudK*	GDP-mannose hydrolase	13.72 (±1.07)
	*rluc*	23S rRNA pseudouridine955/2504/2580 synthase	15.81 (±1.33)
**Repression**	Off-target	–	5.37 (±1.33)
	*rpoC*	DNA-directed RNA polymerase subunit beta'	9.71 (±0.21)
	*yjbG*	Capsule biosynthesis GfcC family protein YjbG	9.99 (±2.88)
	*tig*	Trigger factor	10.88 (±1.98)
	*yhdH*	Acrylyl-CoA reductase (NADPH)	13.27 (±2.07)
	*nlpA*	Lipoprotein-28	14.07 (±0.81)
	*ftsA*	Cell division protein FtsA	16.26 (±1.35)

To explore the potential synergistic effects of the two most effective targets, we simultaneously introduced gRNAs targeting *rluC* and *ftsA* into the violacein-producing strain. The simultaneous regulation of *rluC* and *ftsA* expression led to a remarkable 3.7-fold increase in violacein production (Fig. 3C; Supplementary Table S8; Supplementary Fig. S3), significantly higher than regulating either gene alone. These results suggest that our CRISPRa/i system can effectively modulate complex metabolic pathways across a broad genomic landscape. We anticipate that this approach will facilitate rapid and efficient metabolic engineering in bacteria, potentially accelerating the development of microbial cell factories for diverse biotechnological applications.

## Discussion

Programmable CRISPR-mediated gene regulation on a genome-wide scale has emerged as a powerful tool for controlling metabolic pathways and engineering cellular systems in synthetic biology and industrial biotechnology [1, 2]. While CRISPRi has been shown to effectively modulate gene repression in bacteria, successful applications of CRISPRa remain limited, making the concurrent activation and repression of multiple genes a significant challenge [1]. In this study, we developed an enhanced bacterial dual-mode CRISPRa/i system by combining an evolved PAM-flexible dxCas9 with the *E. coli* CRP effector domain. We showed that our dual-mode CRISPRa/i system can achieve robust activation and repression of target genes, both on plasmids and within the genome.

Facilitated by a pooled gRNA library for genome-wide screening to optimize violacein production in *E. coli*, we significantly enhanced violacein biosynthesis and identified key regulatory targets (*rluC* and *ftsA*), demonstrating the versatility and scalability of our CRISPRa/i system. These results suggest the capability of our system to dissect complex metabolic pathways and identify synergistic regulatory interactions, paving the way for its application in metabolic engineering.

An important consideration for practical applications of bacterial CRISPRa/i systems is their compatibility across bacterial species. Our selection of CRP as an effector domain offers considerable potential for broader applicability, as CRP and its regulatory mechanism are evolutionarily conserved across diverse bacterial species. To demonstrate the compatibility, we implemented our dxCas9–CRP_AR123_ system in *P. putida* KT2440, a biotechnologically relevant and phylogenetically distinct species (Supplementary Fig. S5; Supplementary Note 2). Despite the lower sequence identity between *E. coli* and *P. putida* CRP protein (∼63%), our system exhibited dual-mode functionality, successfully achieving both gene activation and repression [34]. This demonstrates the potential of our system as a broadly applicable platform for bacterial engineering across diverse hosts

Overall, our dual-mode CRISPRa/i system provides a powerful and versatile platform for bacterial engineering, capable of concurrently activating and repressing multiple genes. This system offers significant potential for optimizing metabolic pathways, studying gene networks, and advancing functional genomics in bacterial systems. Furthermore, its cross-species compatibility, flexibility, and scalability make it a promising tool for developing microbial cell factories for a wide range of biotechnological applications, including the production of high-value compounds, biofuels, and pharmaceuticals.

## Supplementary Material

gkaf818_Supplemental_File

## Data Availability

All data are available within the article and its online Supplementary Data.

## Supplementary data

Supplementary data is available at NAR online.
