# Supplementary material for: Dual-mode CRISPRa/i for genome-scale metabolic rewiring in Escherichia coli
Source: Nucleic Acids Res. 2025 Aug 21;53(15):gkaf818. doi: 10.1093/nar/gkaf818 (PMC12370623; doi:10.1093/nar/gkaf818)
Supplement: gkaf818_Supplemental_File [file gkaf818_supplemental_file.pdf]

Supplementary information for:

Dual-mode CRISPRa/i for Genome-Scale Metabolic Rewiring in *Escherichia coli*

Soo Young Moon[a,b], Mi Ri Kim[c], Nan Yeong An[a], Myung Hyun Noh[c,*], Ju Young Lee[a,d,*]

[a] Department of Biological Sciences, Korea Advanced Institute of Science and Technology (KAIST), Daejeon, 34141, Republic of Korea
[b] School of Interdisciplinary Bioscience and Bioengineering, Pohang University of Science and Technology (POSTECH), Pohang, Gyeongbuk 37673, Republic of Korea
[c] Research Center for Bio-Based Chemistry, Korea Research Institute of Chemical Technology (KRICT), Ulsan 44429, Republic of Korea
[d] Graduate School of Engineering Biology, Korea Advanced Institute of Science and Technology (KAIST), Daejeon, 34141, Republic of Korea

[*]Corresponding authors:

Prof. Ju Young Lee
Phone: +82-42-350-2629
E-mail: juyounglee@kaist.ac.kr

Dr. Myung Hyun Noh
Phone: +82-52-241-6362
Fax: +82-52-241-6359
E-mail: mhnoh@krict.re.kr

__**Table of Contents**__

Supplementary Table S1. Comparison of CRISPRa/i system in *E. coli*

Supplementary Table S2. Strains and plasmids used in this study.

Supplementary Table S3. Primers used in this study.

Supplementary Table S4. DNA sequences of CRP derivatives used in this study.

Supplementary Table S5. gRNAs targeting reporter genes.

Supplementary Table S6. gRNAs involved in regulating violacein production.

Supplementary Table S7. Genes influencing violacein production through the CRISPRa/i system.

Supplementary Table S8. Synergistic increase in violacein production through simultaneous activation of *rluC* and repression of *ftsA*.

Supplementary Figure S1. CRISPRa/i target region information and gRNA sequences for reporter gene targeting.

Supplementary Figure S2. Growth curves of *E. coli* strains expressing dxCas9 or dxCas9–CRP derivatives compared to wild-type (WT) *E. coli*.

Supplementary Figure S3. Transcriptional changes of CRISPRa/i-targeted genes in violacein-producing *E. coli* strains.

Supplementary Figure S4. CRISPRa driven by dxCas9 combined with cAMP-independent CRP mutants (dxCas9-CRP_{mutant}).

Supplementary Figure S5. Simultaneous activation and repression of multiple genes using the CRISPRa/i system in *P. putida*

Supplementary Note 1. CRP as the effector domain for CRISPRa.

Supplementary Note 2. Compatibility of the dxCas9-CRP system in *P. putida* KT2440.

Supplementary Materials and Methods. RT-qPCR analysis.

Supplementary Table S1. Comparison of CRISPRa/i system in *E. coli*

DNA binding domain of Cas9	Effector domain	Recruitment strategy	sgRNA	Target genes (plasmid or genome)	Genome-scale reprogramming	Cross-species utility	Reference
dxCas9	*E. coli* CRP variants	Directly fusing activator domain to dxCas9	Genome-wide sgRNA	GFP and mCherry reporter genes on plasmids; genome	Yes	*E. coli, P. putida*	Our study
dCas9	*E. coli* $SoxS_{R93A/S101A}$	Recruiting activator domain to dCas9 using MC2 coat protein (MSP)	Designed sgRNAs with MS2 scaffold	GFP and mCherry reporter genes on plasmids	No	*E. coli*	[1] Fontana et al. (2020)
dxCas9	*E. coli* bEBPs (PspF)	Recruiting activator domain to dCas9 using RNA binding domain (λN22plus)	Designed sgRNAs with BoxB scaffold	sfGFP reporter genes on plasmids	No	*E. coli, Klebsiella oxytoca*	[2] Liu et al. (2019)
			Designed sgRNAs library with BoxB scaffold	*vioA*, *vioB*, *vioD*, *vioE*, *vioC* genes on plasmid (violacein production)			
dCas9	*E. coli* $SoxS_{R93A}$	Recruiting activator domain to dCas9 using MC2 coat protein (MSP)	Designed sgRNAs with MS2 scaffold	GFP and mCherry reporter genes on plasmids	No	*E. coli*	[3] Dong et al. (2018)
				pdc adhB gene cassette on plasmid (ethanol production)			
dCas9	T4 bacteriophage AsiA	Directly fusing activator domain to dCas9	Single sgRNA	GFP and mCherry reporter genes on plasmids	No	*E. coli*, *Streptococcus pneumoniae*	[4] Bikard et al. (2013)
dCas9	*E. coli* RpoZ						

Supplementary Table S2. Strains and plasmids used in this study.

**Strains**		
E. coli MG1655 (DE3)	*E. coli* MG1655 ΔendA ΔrecA (DE3)	Addgene strain
P. putida KT2440	*P. putida* KT2440 wild-type	ATCC 47054
**Plasmids**		
	***E. coli* dual-mode CRISPRa/i system**	
**dxCas9-CRP variants plasmids**	**Description / Genotype**	**Reference**
dxCas9(3.7)-VPR	pCMV vector containing dxCas9(3.7)-NLS-VP64-p65-Rta, Amp^{R}	Addgene plasmid
pACCRi	pACYC-based vector containing $_{rha}P_{BAD}$-*dCas9*, Cm^{R}, p15A ori	[5]
pdxCas9	pACCRi vector containing $_{rha}P_{BAD}$-*dxCas9*, Cm^{R}, p15A ori	This study
pdxCas9-L10-CRP$_{WT}$	pdxCas9 vector containing linker (10 aa) with CRP$_{WT}$	This study
pdxCas9-L10-CRP$_{AR123}$	pdxCas9 vector containing linker (10 aa) with CRP$_{AR123}$	This study
pdxCas9-L10-CRP$_{AR23}$	pdxCas9 vector containing linker (10 aa) with CRP$_{AR23}$	This study
pdxCas9-L10-CRP$_{AR1}$	pdxCas9 vector containing linker (10 aa) with CRP$_{AR1}$	This study
pdxCas9-L10-CRP$_{AR3}$	pdxCas9 vector containing linker (10 aa) with CRP$_{AR3}$	This study
**Reporter plasmid**	**Description / Genotype**	**Reference**
pMW7	P$_{T7}$-GFP, pMB1 ori, Amp^{R}	[5], [6]
pA-P$_{J23117}$-GFP	pMW7 vector containing BBa_J23117-GFP, pMB1 ori, Amp^{R}	This study
pR-P$_{J23119}$-GFP	pMW7 vector containing BBa_J23119-GFP, pMB1 ori, Amp^{R}	This study
pP$_{J23117}$-GFP-P$_{J23119}$-mCherry	pMW7 vector containing BBa_J23117-GFP, BBa_J23119-mCherry, pMB1 ori, Amp^{R}	This study
**gRNA plasmid**	**Description / Genotype**	**Reference**
psgRNA	BBa_J23119-sgRNA, ColE1 ori, Kan^{R}	Addgene plasmid
psgRNA (off-target)	psgRNA vector containing BBa_J23119, sgRNA (off-target) expression cassette, ColE1 ori, Kan^{R}	This study
psgRNA (A1-A13)	psgRNA vector containing BBa_J23119, sgRNA (A1-A13) expression cassette, ColE1 ori, Kan^{R}	This study
psgRNA (R1-R4)	psgRNA vector containing BBa_J23119, sgRNA (R1-R4) expression cassette, ColE1 ori, Kan^{R}	This study
psgRNA (A7/R4)	psgRNA vector containing BBa_J23119-sgRNA (A7) / BBa_J23119-sgRNA (R4) expression cassette, ColE1 ori, Kan^{R}	This study
psgRNA (rluC)	psgRNA vector containing constitutive sgRNA (rluC) expression cassette, ColE1 ori, Kan^{R}	This study
psgRNA (ftsA)	psgRNA vector containing constitutive sgRNA (ftsA) expression cassette, ColE1 ori, Kan^{R}	This study
psgRNA (rluC-ftsA)	psgRNA vector containing constitutive sgRNA (rluC-ftsA) expression cassette, ColE1 ori, Kan^{R}	This study
**Violacein production plasmid**	**Description / Genotype**	**Reference**
pVio	pET vector containing P$_{T7}$-vioA-P$_{T7}$-vioB-P$_{T7}$-vioC-P$_{T7}$-vioD-P$_{T7}$-vioE, pMB1 ori, Amp^{R}	Addgene plasmid

P. putida dual-mode CRISPRa/i system		
pPP_dualMode_off	P_{Lac}-dxCas9_CRP$_{AR123}$, BBa_J23117-GFP, BBa_J23119-mCherry, BBa_J23119-sgRNA (off-target) BBR1 ori, Km^R	This study
pPP_dualMode_A9	P_{Lac}-dxCas9_CRP$_{AR123}$, BBa_J23117-GFP, BBa_J23119-mCherry, BBa_J23119-sgRNA (A9) BBR1 ori, Km^R	This study
pPP_dualMode_R4	P_{Lac}-dxCas9_CRP$_{AR123}$, BBa_J23117-GFP, BBa_J23119-mCherry, BBa_J23119-sgRNA (R4) BBR1 ori, Km^R	This study
pPP_dualMode_A9_R4	P_{Lac}-dxCas9_CRP$_{AR123}$, BBa_J23117-GFP, BBa_J23119-mCherry, BBa_J23119-sgRNA (A9/R4) BBR1 ori, Km^R	This study

Supplementary Table S3. Primers in this study.

Primer name	Primer sequence (5'-3')
dxCas9-NdeI-F	agcaggatcaccatatggacaagaagtactccattgggctc
dxCas9-linker-SpeI-R	ctggaagaggactagtgccggcgccgccgccgtctccaccgagctgagagag
linker10-CRP-NgoMIV-F	cggcggcggcgccggcggcggcggcgccggcgtgcttggcaaaccgcaaac
CRP-NgoMIV-R	gaggactagtgccggttagtggtggtggtggtggtgacgagtgccgtaaacgacga
CRP_{AR123}-NgoMIV-R	gaggactagtgccggttagtggtggtggtggtggtgagaacagccgacaatctgacca
CRP_{AR23}-NgoMIV-R	gaggactagtgccggttagtggtggtggtggtggtggtcgaggaacgccaggttg
linker10-CRP_{AR1}-NgoMIV-F	cggcggcggcgccggcggcggcggcgccggcgcgttcctcgacgtgacgg
linker10-CRP_{AR3}-NgoMIV-F	cggcggcggcgccggcggcggcggcgccggcagcacgcttattcaccaggg
CRP_{AR3}-NgoMIV-R	gaggactagtgccggttagtggtggtggtggtggtgggcggttttcgcacgtacc
Linker5-CRP-NgoMIV-F	cggcggcggcgccggcgtgcttggcaaaccgcaaac
Linker20-CRP-NgoMIV-F	cggcggcggcgccggcggcggcggcgccggcggcggcggcgccggcggcggcggcgccggcgtgctt
J1-NdeI-F	aaggagatatacatatggcctacggtatccaccgg
BBa_J23117-R	tcttctcctttactcatatgacctttctcctctttaatgaattcgctagcacaatccctaggactgagctagctgtcaagtcc
BBa_J23119-R	tcttctcctttactcatatgacctttctcctctttaatgaat
mCherry-AatII-F	ataatggtttcttaggcggccgcggatccttgacagctagctcagtcctaggtat
mCherry-AatII-R	gaaaagtgccacctgactagtgccggccaaaaaacccctcaagacccg
sgRNA(A1)-F	gtcctaggtataatactagtagtgagtcgtattaatttcggttttagagctagaaatagcaagtt
sgRNA(A2)-F	gtcctaggtataatactagttcactatagggagaccacaagttttagagctagaaatagcaagtt
sgRNA(A3)-F	gtcctaggtataatactagtttaaacaaaattatttctaggttttagagctagaaatagcaagtt
sgRNA(A4)-F	gtcctaggtataatactagtaattttgtttaactttaagagttttagagctagaaatagcaagtt
sgRNA(A5)-F	gtcctaggtataatactagtttaagaaggagatataaatagttttagagctagaaatagcaagtt
sgRNA(A6)-F	gtcctaggtataatactagtatatggcctacggtatccacgttttagagctagaaatagcaagtt
sgRNA(A7)-F	gtcctaggtataatactagtaggctgccataggtctccgggttttagagctagaaatagcaagtt
sgRNA(A8)-F	gtcctaggtataatactagtcaaaggtgtcctatggcggcgttttagagctagaaatagcaagtt
sgRNA(A9)-F	gtcctaggtataatactagtaggtcaccccttggcaaccaagttttagagctagaaatagcaagtt
sgRNA(A10)-F	gtcctaggtataatactagtagggtgacctatggtgaccagttttagagctagaaatagcaagtt
sgRNA(A11)-F	gtcctaggtataatactagttggtaaccgcaggacaccgcgttttagagctagaaatagcaagtt
sgRNA(A12)-F	gtcctaggtataatactagtaggacgcctttggtaaccgcgttttagagctagaaatagcaagtt
sgRNA(A13)-F	gtcctaggtataatactagtcggtgtcctgcggttaccaagttttagagctagaaatagcaagtt
sgRNA(R1)-F	taatactagtttgacagctagctcagtcctgttttagagctagaaatagcaagtt
sgRNA(R2)-F	taatactagtcatctaattcaacaagaattgttttagagctagaaatagcaagtt
sgRNA(R3)-F	taatactagtagtagtgcaaataaatttaagttttagagctagaaatagcaagtt
sgRNA(R4)-F	taatgctagcttcttcaccccttacttaccagttttagagctagaaatagcaagtt
gRNA(R4)-ppuMI-F	cactttacgggtcctttgacagctagctcagtcctag
gRNA(R4)-ppuMI-R	caccggaaaggacccatttgtcctactcaggagagcg
gRNA_rluC-F	tgctataacaaggcttgcaggttttagagctagaaatagcaagttaaaataaggc
gRNA_ftsA-F	gctctcatcttttccaacgagttttagagctagaaatagcaagttaaaataaggc
psgRNA-J23119p-R	actagtattatacctaggactgagctagc
J23119_ApaI_ftsA_F	gccctttcgtcttcgggcccttgacagctagctcagtcctaggt
ftsA_ApaI_rrnB_T1-R	gcacagaacttaatgggcccatttgtcctactcaggagagcgttc
pPP-BBR1-F	ctttgcgttccgtttgcacccagtg
pPP-BBR1-R	cactgggtgcaaacggaacgcaaag
pPP-CRPA123-F	gcagaacagtcaaaatggggacgtggatatggacaagaagtactccattgggct
pPP-CRPA123-R	ggcaggctatttaataacggcgttattagtggtggtggtggtggtgagaa

pPP-CRPA123-R2	cagatggcaggctatttaataacggcgttagaaggagctgactgggttgaaggctctc
pPP-F2	ttctcaccaccaccaccaccactaataacgccgttattaaatagcctgcc
pPP-GFP-F	agaacgcagaagcggtctgataaaagcctacggtatccaccggagacctatggca
pPP-GFP-R	gcgctactgccgccaggcaaattctggatccaaaaaacccctcaagaccc
pPP-gRNA(off)-R	ggcaggctatttaataacggcgttaagctagtgccggttagtggtggtggt
pPP-gRNA(R4)-F	ttgacagctagctcagtcctaggtataatgctagcttcttcacccttacttacca
pPP-gRNA(R4)-R	actagtattatacctaggactgagctagctgtcaaagatctttaaccggtcgccgagg
pPP-R	tgccataggtctccggtggataccgtaggcttttatcagaccgcttctgcgttct
pPP-R	gggtcttgaggggttttttggatccagaatttgcctggcggcagtagcgc
pPP-R2	agcccaatggagtacttcttgtccatatccacgtccccattttgactgttctgc
pPP-RBS-R	atccacgtccccattttgactgttctgc
pPP-Ter-F	taacgccgttattaaatagcctgcc
pPP-BBR1-F	ctttgcgttccgtttgcacccagtg
pPP-BBR1-R	cactgggtgcaaacggaacgcaaag

Supplementary Table S4. DNA sequences of CRP derivatives used in this study.

CRP derivates	Sequence
Wild-type CRP	GTGCTTGGCAAACCGCAAACAGACCCGACTCTCGAATGGTTCTTGTCTCATTGCCACATT<br>CATAAGTACCCATCCAAGAGCACGCTTATTCACCAGGGTGAAAAAGCGGAAACGCTGTAC<br>TACATCGTTAAAGGCTCTGTGGCAGTGCTGATCAAAGACGAAGAGGGTAAAGAAATGATC<br>CTCTCCTATCTGAATCAGGGTGATTTTATTGGCGAACTGGGCCTGTTTGAAGAGGGCCAG<br>GAACGTAGCGCATGGGTACGTGCGAAAACCGCCTGTGAAGTGGCTGAAATTTCGTACAAA<br>AAATTTCGCCAATTGATTCAGGTAAACCCGGACATTCTGATGCGTTTGTCTGCACAGATG<br>GCGCGTCGTCTGCAAGTCACTTCAGAGAAAGTGGGCAACCTGGCGTTCCTCGACGTGACG<br>GGCCGCATTGCACAGACTCTGCTGAATCTGGCAAAACAACCAGACGCTATGACTCACCCG<br>GACGGTATGCAAATCAAAATTACCCGTCAGGAAATTGGTCAGATTGTCGGCTGTTCTCGT<br>GAAACCGTGGGACGCATTCTGAAGATGCTGGAAGATCAGAACCTGATCTCCGCACACGGT<br>AAAACCATCGTCGTTTACGGCACTCGT
CRP_{AR123}	GTGCTTGGCAAACCGCAAACAGACCCGACTCTCGAATGGTTCTTGTCTCATTGCCACATT<br>CATAAGTACCCATCCAAGAGCACGCTTATTCACCAGGGTGAAAAAGCGGAAACGCTGTAC<br>TACATCGTTAAAGGCTCTGTGGCAGTGCTGATCAAAGACGAAGAGGGTAAAGAAATGATC<br>CTCTCCTATCTGAATCAGGGTGATTTTATTGGCGAACTGGGCCTGTTTGAAGAGGGCCAG<br>GAACGTAGCGCATGGGTACGTGCGAAAACCGCCTGTGAAGTGGCTGAAATTTCGTACAAA<br>AAATTTCGCCAATTGATTCAGGTAAACCCGGACATTCTGATGCGTTTGTCTGCACAGATG<br>GCGCGTCGTCTGCAAGTCACTTCAGAGAAAGTGGGCAACCTGGCGTTCCTCGACGTGACG<br>GGCCGCATTGCACAGACTCTGCTGAATCTGGCAAAACAACCAGACGCTATGACTCACCCG<br>GACGGTATGCAAATCAAAATTACCCGTCAGGAAATTGGTCAGATTGTCGGCTGTTCT
CRP_{AR23}	GTGCTTGGCAAACCGCAAACAGACCCGACTCTCGAATGGTTCTTGTCTCATTGCCACATT<br>CATAAGTACCCATCCAAGAGCACGCTTATTCACCAGGGTGAAAAAGCGGAAACGCTGTAC<br>TACATCGTTAAAGGCTCTGTGGCAGTGCTGATCAAAGACGAAGAGGGTAAAGAAATGATC<br>CTCTCCTATCTGAATCAGGGTGATTTTATTGGCGAACTGGGCCTGTTTGAAGAGGGCCAG<br>GAACGTAGCGCATGGGTACGTGCGAAAACCGCCTGTGAAGTGGCTGAAATTTCGTACAAA<br>AAATTTCGCCAATTGATTCAGGTAAACCCGGACATTCTGATGCGTTTGTCTGCACAGATG<br>GCGCGTCGTCTGCAAGTCACTTCAGAGAAAGTGGGCAACCTGGCGTTCCTCGAC
CRP_{AR1}	GCGTTCCTCGACGTGACGGGCCGCATTGCACAGACTCTGCTGAATCTGGCAAAACAACCA<br>GACGCTATGACTCACCCGGACGGTATGCAAATCAAAATTACCCGTCAGGAAATTGGTCAG<br>ATTGTCGGCTGTTCT
CRP_{AR3}	AGCACGCTTATTCACCAGGGTGAAAAAGCGGAAACGCTGTACTACATCGTTAAAGGCTCT<br>GTGGCAGTGCTGATCAAAGACGAAGAGGGTAAAGAAATGATCCTCTCCTATCTGAATCAG<br>GGTGATTTTATTGGCGAACTGGGCCTGTTTGAAGAGGGCCAGGAACGTAGCGCATGGGTA<br>CGTGCGAAAACCGCC

Supplementary Table S5. gRNAs targeting reporter genes.

sgRNA	Sequence	Target	Target Strand[a]	Distance to TSS[b]
sgRNA (Off-target)[c]	CCTCTTCCAGTTAGTAAATC	-	-	-
sgRNA (A1)	AGTGAGTCGTATTAATTTCG	GFP	NT	-294
sgRNA (A2)	TTTCTAGAGGGAAACCGTTG	GFP	NT	-262
sgRNA (A3)	TCACTATAGGGAGACCACAA	GFP	NT	-249
sgRNA (A4)	AATTTTGTTTAACTTTAAGA	GFP	T	-222
sgRNA (A5)	TTAAGAAGGAGATATAAATA	GFP	T	-208
sgRNA (A6)	AGGCTGCCATAGGTCTCCGG	GFP	T	-191
sgRNA (A7)	AGGGTGACCTATGGTGACCA	GFP	NT	-191
sgRNA (A8)	CAAAGGTGTCCTATGGCGGC	GFP	NT	-168
sgRNA (A9)	AGGTCACCCTTGGCAACCAA	GFP	NT	-151
sgRNA (A10)	AGGGTGACCTATGGTGACCA	GFP	NT	-141
sgRNA (A11)	TGGTAACCGCAGGACACCGC	GFP	NT	-91
sgRNA (A12)	AGGACGCCTTTGGTAACCGC	GFP	NT	-81
sgRNA (A13)	CGGTGTCCTGCGGTTACCAA	GFP	T	-71
sgRNA (R1)	TTGACAGCTAGCTCAGTCCT	GFP	T	-15
sgRNA (R2)	CATCTAATTCAACAAGAATT	GFP	NT	+66
sgRNA (R3)	AGTAGTGCAAATAAATTTAA	GFP	NT	+158
sgRNA (R4)	TTCTTCACCCTTACTTACCA	mCherry	NT	+28

[a] Template strand (T) or non-template strand (NT).

[b] Distance to TSS is the distance from the 3' end (PAM proximal) of the guide target site to the transcription start site (TSS). The TSS corresponds to the first nucleotide of the ribosome binding site immediately downstream of the *BBa* promoter sequence (see complete maps below Supplementary Fig. S1).

[c] In this study, we employed the same off-target gRNA that was previously used as a negative control in *E. coli* CRISPRi experiments by Kim et al. [5].

Supplementary Table S6. gRNAs involved in regulating violacein production.

**sgRNA**	**Sequence**	**Target**
sgRNA (*gstA*)	CAGCATCAGTACTGCAATAA	*gstA*
sgRNA (*rplY*)	TTTCACGCACCTGCCAGTAC	*rplY*
sgRNA (*xerD*)	GGTGACCTTACATAACCTCA	*xerD*
sgRNA (*yfhH*)	ATCAGGATGTTTCAGTCCAG	*yfhH*
sgRNA (*alaA*)	CTGATTCTTAATTGTAAGTC	*alaA*
sgRNA (*rluC*)	TGCTATAACAAGGCTTGCAG	*rluC*
sgRNA (*rlmG*)	ACGCCACCGATAAAGCCC	*rlmG*
sgRNA (*yqgE*)	TCAATCTCACGAATACAGGT	*yqgE*
sgRNA (*rppH*)	TCAATCTCACGAATACAGGT	*rppH*
sgRNA (*ilvY*)	CTGGGCAAATGTCGCTTTAT	*ilvY*
sgRNA (*lysO*)	CCCACATGGATGTGGGCTGA	*lysO*
sgRNA (*nudK*)	TGGTGGTCTGGTAACGGTAC	*nudK*
sgRNA (*ushA*)	CTGAAAAAGTGACTTTCCTA	*ushA*
sgRNA (*ydeE*)	GGTAGAATGTCGCGAAAAGA	*ydeE*
sgRNA (*opgC*)	TTCTGTCTTTAGGAGAAGCA	*opgC*
sgRNA (*basR*)	CTATCCGGGGCGATGGCATA	*basR*
sgRNA (*yhdH*)	GCTCCGCCAGCCCACCCCAG	*yhdH*
sgRNA (*mlaA*)	GGTTCAGTCCGTTGCAGTTT	*mlaA*
sgRNA (*cusA*)	GGTGGCGGTTTCCGCTTTCC	*cusA*
sgRNA (*kbl*)	GGAGTCGTCTACCATCACCA	*kbl*
sgRNA (*alaW*)	AAGCTTGTCGAGTTGACGGG	*alaW*
sgRNA (*arsB*)	CAGCGCGCCCACAGCTGCAA	*arsB*
sgRNA (*ftp*)	CGCGCCAATGCGCAGCGAGG	*ftp*
sgRNA (*yjbG*)	CTCTTCGCTAATCACCGCAC	*yjbG*
sgRNA (*sad*)	TATCGAAAACGCACTTCAGC	*sad*
sgRNA (*ybiW*)	GTGTGGGCTTTACGCCCGTC	*ybiW*
sgRNA (*mhpA*)	GCGACGCCTTTAACCTCGCA	*mhpA*
sgRNA (*der*)	CGACGAAGACGCTGAATACT	*der*
sgRNA (*rpoC*)	TTTACGCTTGGTTTCGGAGT	*rpoC*
sgRNA (*ftsA*)	GCTCTCATCTTTTCCAACGA	*ftsA*
sgRNA (*rplB*)	GGTGGTGAAGGTCGTAACTT	*rplB*
sgRNA (*spoT*)	TGTCCGTGGCTTTGGGTTGC	*spoT*
sgRNA (*nlpA*)	ACGGTGGCGCGGTGCCTGGC	*nlpA*
sgRNA (*tig*)	TCACCCAGCTTGTATTCGCC	*tig*

Supplementary Table S7. Genes influencing violacein production through the CRISPRa/i system.

gRNA	Functional classification		Function	Targeted gene	Violacein (mg/L)	Violacein production relative to an off-target control
Activation			-	off-target	5.37 (±1.33)	1
	Cellular processes	Lysosome	Glutathione S-transferase	*gstA*	0.47 (±0.08)	0.1
	Environmental information processing	ABC transporters	Uncharacterized HTH-type transcriptional regulator	*yfhH*	3.80 (±1.50)	0.7
		Two-component system	Integrase/Recombinase	*xerD*	4.16 (±1.33)	0.8
	Genetic information processing	Chromosome and associated proteins	Large subunit ribosomal protein L25	*rplY*	6.39 (±2.72)	1.2
		Ribosome	23S rRNA (guanine1835-N2)-methyltransferase	*rlmG*	6.99 (±0.16)	1.3
		Ribosome biogenesis	L-lysine/thialysine efflux transporter	*lysO*	9.26 (±1.88)	1.7
		Transcription factors	5'-nucleotidase / UDP-sugar diphosphatase	*ushA*	9.58 (±0.40)	1.8
		RNA degradation	LysR family transcriptional regulator, positive regulator for ilvC	*ilvY*	9.68 (±3.83)	1.8
		Ribosome biogenesis	Alanine-synthesizing transaminase	*alaA*	9.90 (±0.51)	1.8
	Metabolism	Glutathione metabolism	Putative (di)nucleoside polyphosphate hydrolase	*rppH*	10.01 (±0.35)	1.9
		Purine metabolism	Molybdate transport system ATP-binding protein	*opgC*	10.88 (±2.26)	2.0
		Alanine, aspartate and glutamate metabolism	Putative transcriptional regulator	*ydeE*	12.27 (±1.20)	2.3
		Enzymes with EC numbers	OmpR family, response regulator BasR	*basR*	12.72 (±0.60)	2.4
	Signaling and cellular processes	Transporters	MFS transporter, YQGE family, putative transporter	*yqgE*	13.12 (±1.76)	2.4
	Unknown	-	GDP-mannose hydrolase	*nudK*	13.72 (±1.07)	2.6
		-	23S rRNA pseudouridine955/2504/2580 synthase	*rluC*	15.81 (±1.33)	2.9
Repression			-	Off target	5.37 (±1.33)	1
	Cellular processes	Cell cycle - Caulobacter	Cell division protein FtsA	*ftsA*	16.26 (±1.35)	3.0
	Environmental information processing	ABC transporters	copper/silver efflux system protein	*cusA*	2.20 (±6.89)	0.4
		Two-component system	Lipoprotein-28	*nlpA*	14.07 (±0.81)	2.6

Genetic information processing	Ribosome biogenesis	GTP-binding protein EngA	*der*	2.18 (±0.50)	0.4
	Ribosome	large subunit ribosomal protein L2	*rplB*	6.89 (±0.79)	1.3
	Protein processing	Succinate-semialdehyde dehydrogenase	*sad*	7.03 (±0.47)	1.3
	Ribosome biogenesis	Aminoacyl-tRNA biosynthesis, tRNA-Ala	*alaW*	8.49 (±0.55)	1.6
	Aminoacyl-tRNA biosynthesis	DNA-directed RNA polymerase subunit beta'	*rpoC*	9.71 (±0.21)	1.8
	RNA polymerase	Trigger factor	*tig*	10.88 (±1.98)	2.0
Metabolism	Butanoate metabolism	3-(3-hydroxy-phenyl) propionate hydroxylase	*mhpA*	7.11 (±1.63)	1.3
	Phenylalanine metabolism	Glycine C-acetyltransferase	*kbl*	7.84 (±0.92)	1.5
	Glycine, serine and threonine metabolism	GTP diphosphokinase / guanosine-3',5'-bis(diphosphate) 3'-diphosphatase	*spoT*	8.14 (±2.11)	1.5
	Enzymes with EC numbers	FAD:protein FMN transferase	*ftp*	8.61 (±0.33)	1.6
	Propanoate metabolism	Acrylyl-CoA reductase (NADPH)	*yhdH*	13.27 (±2.07)	2.5
Signaling and cellular processes	Transporters	Arsenical pump membrane protein	*arsB*	1.35 (±0.41)	0.3
	Structural proteins	Phospholipid-binding lipoprotein MlaA	*mlaA*	9.16 (±1.25)	1.7
Unknown	-	Putative pyruvate formate lyase	*ybiW*	1.75 (±0.87)	0.3
	-	Capsule biosynthesis GfcC family protein YjbG	*yjbG*	9.99 (±2.88)	1.9

Supplementary Table S8. Synergistic increase violacein production through simultaneous activation of *rluC* and repression of *ftsA*.

**gRNA**	**Targeted gene**	**OD_{600}**	**Violacein (mg/L)**	**Violacein production relative to an off-target control**
-	off-target	14.50 (±0.29)	5.37 (±1.33)	1
Activation	*rluC*	14.55 (±0.78)	15.81 (±1.33)	2.9
Repression	*ftsA*	14.85 (±0.35)	16.26 (±1.35)	3.0
Dual mode regulation	*rluC/ftsA*	14.05 (±0.07)	19.85(±0.54)	3.7

Supplementary Figure S1. CRISPRa/i target region information and gRNA sequences for reporter gene targeting.

Weak promoter-GFP Reporter (Figure 2B-2D)

Annotations: Upstream region, BBa_J23117 promoter, RBS, GFP

CCCGCGAAATTAATACGACTCACTATAGGGAGACCACAACGGTTTCCCTCTAGAAATAATTTTGTTTA
ACTTTAAGAAGGAGATATACATATGGCCTACGGTATCCACCGGAGACCTATGGCAGCCTCCGGCCGCC
ATAGGACACCTTTGGTTGCCAAGGGTGACCTATGGTGACCATGGGCCACCACGGGCGACCTCAGGTAT
CCTGCGGTGTCCTGCGGTTACCAAAGGCGTCCTTTGGGTTCCACCGGATACCTCCGGACTTGACAGCT
AGCTCAGTCCTAGGGATTGTGCTAGCGAATTCATTAAAGAGGAGAAAGGTCATATGAGTAAAGGAGAA
GAACTTTTCACTGGAGTTGTCCCAATTCTTGTTGAATTAGATGGTGATGTTAATGGGCACAAATTTTC
TGTCAGTGGAGAGGGTGAAGGTGATGCAACATACGGAAAACTTACCCTTAAATTTATTTGCACTACTG
GAAAACTACCTGTTCCATGGCCAACACTTGTCACTACTCTGACCTATGGTGTTCAATGCTTTTCCCGT
TATCCGGATCACATGAAACGGCATGACTTTTTCAAGAGTGCCATGCCCGAAGGTTATGTACAGGAACG
CACTATATCTTTCAAAGATGACGGGAACTACAAGACGCGTGCTGAAGTCAAGTTTGAAGGTGATACCC
TTGTTAATCGTATCGAGTTAAAAGGTATTGATTTTAAAGAAGATGGAAACATTCTCGGACACAAACTC
GAGTACAACTATAACTCACACAATGTATACATCACGGCAGACAAACAAAAGAATGGAATCAAAGCTAA
CTTCAAAATTCGCCACAACATTGAAGATGGTTCCGTTCAACTAGCAGACCATTATCAACAAAATACTC
CAATTGGCGATGGCCCTGTCCTTTTACCAGACAACCATTACCTGTCGACACAATCTGCCCTTTCGAAA
GATCCCAACGAAAAGCGTGACCACATGGTCCTTCTTGAGTTTGTAACTGCTGCTGGGATTACACATGG
CATGGATGAGCTCTACAAATAA

Strong promoter-GFP Reporter (Figure 2F)

Annotations: Upstream region, BBa_J23119 promoter, RBS, GFP

CCCGCGAAATTAATACGACTCACTATAGGGAGACCACAACGGTTTCCCTCTAGAAATAATTTTGTTTA
ACTTTAAGAAGGAGATATACATATGGCCTACGGTATCCACCGGAGACCTATGGCAGCCTCCGGCCGCC
ATAGGACACCTTTGGTTGCCAAGGGTGACCTATGGTGACCATGGGCCACCACGGGCGACCTCAGGTAT
CCTGCGGTGTCCTGCGGTTACCAAAGGCGTCCTTTGGGTTCCACCGGATACCTCCGGACTTGACAGCT
AGCTCAGTCCTAGGTATAATGCTAGCGAATTCATTAAAGAGGAGAAAGGTCATATGAGTAAAGGAGAA
GAACTTTTCACTGGAGTTGTCCCAATTCTTGTTGAATTAGATGGTGATGTTAATGGGCACAAATTTTC
TGTCAGTGGAGAGGGTGAAGGTGATGCAACATACGGAAAACTTACCCTTAAATTTATTTGCACTACTG
GAAAACTACCTGTTCCATGGCCAACACTTGTCACTACTCTGACCTATGGTGTTCAATGCTTTTCCCGT
TATCCGGATCACATGAAACGGCATGACTTTTTCAAGAGTGCCATGCCCGAAGGTTATGTACAGGAACG
CACTATATCTTTCAAAGATGACGGGAACTACAAGACGCGTGCTGAAGTCAAGTTTGAAGGTGATACCC
TTGTTAATCGTATCGAGTTAAAAGGTATTGATTTTAAAGAAGATGGAAACATTCTCGGACACAAACTC
GAGTACAACTATAACTCACACAATGTATACATCACGGCAGACAAACAAAAGAATGGAATCAAAGCTAA
CTTCAAAATTCGCCACAACATTGAAGATGGTTCCGTTCAACTAGCAGACCATTATCAACAAAATACTC
CAATTGGCGATGGCCCTGTCCTTTTACCAGACAACCATTACCTGTCGACACAATCTGCCCTTTCGAAA
GATCCCAACGAAAAGCGTGACCACATGGTCCTTCTTGAGTTTGTAACTGCTGCTGGGATTACACATGG
CATGGATGAGCTCTACAAATAA

Weak promoter-GFP-Strong promoter-mCherry Reporter (Figure 2H)

Annotations: Upstream region, BBa_J23117 promoter, BBa_J23119 promoter, RBS, GFP, mCherry

GCCTACGGTATCCACCGGAGACCTATGGCAGCCTCCGGCCGCCATAGGACACCTTTGGTTGCCAAGGG
TGACCTATGGTGACCATGGGCCACCACGGGCGACCTCAGGTATCCTGCGGTGTCCTGCGGTTACCAAA
GGCGTCCTTTGGGTTCCACCGGATACCTCCGGACTTGACAGCTAGCTCAGTCCTAGGGATTGTGCTAG
CGAATTCATTAAAGAGGAGAAAGGTCATATGAGTAAAGGAGAAGAACTTTTCACTGGAGTTGTCCCAA
TTCTTGTTGAATTAGATGGTGATGTTAATGGGCACAAATTTTCTGTCAGTGGAGAGGGTGAAGGTGAT
GCAACATACGGAAAACTTACCCTTAAATTTATTTGCACTACTGGAAAACTACCTGTTCCATGGCCAAC
ACTTGTCACTACTCTGACCTATGGTGTTCAATGCTTTTCCCGTTATCCGGATCACATGAAACGGCATG
ACTTTTTCAAGAGTGCCATGCCCGAAGGTTATGTACAGGAACGCACTATATCTTTCAAAGATGACGGG
AACTACAAGACGCGTGCTGAAGTCAAGTTTGAAGGTGATACCCTTGTTAATCGTATCGAGTTAAAAGG
TATTGATTTTAAAGAAGATGGAAACATTCTCGGACACAAACTCGAGTACAACTATAACTCACACAATG
TATACATCACGGCAGACAAACAAAAGAATGGAATCAAAGCTAACTTCAAAATTCGCCACAACATTGAA
GATGGTTCCGTTCAACTAGCAGACCATTATCAACAAAATACTCCAATTGGCGATGGCCCTGTCCTTTT
ACCAGACAACCATTACCTGTCGACACAATCTGCCCTTTCGAAAGATCCCAACGAAAAGCGTGACCACA
TGGTCCTTCTTGAGTTTGTAACTGCTGCTGGGATTACACATGGCATGGATGAGCTCTACAAATAATAA
GGTACCATGGTAAGCTTAGGCCTCTAGTCTAGACTAGAATTCCGATCCGGCTGCTAACAAAGCCCGAA
AGGAAGCTGAGTTGGCTGCTGCCACCGCTGAGCAATAACTAGCATAACCCCTTGGGGCCTCTAAACGG
GTCTTGAGGGGTTTTTTGCTGAAAGGAGGAACTATATCCGGATAATTCTTGAAGACGAAAGGGCCTCG
TGATACGCCTATTTTTATAGGTTAATGTCATGATAATAATGGTTTCTTAGGCGGCCGCGGATCCTTGA
CAGCTAGCTCAGTCCTAGGTATAATTGCTAAGCGAATTCCATTAAAGAGGAGAAAGGTACCATGGTAA
GTAAGGGTGAAGAAGACAATATGGCGATCATTAAGGAATTCATGCGTTTCAAAGTACACATGGAGGGA
AGCGTGAACGGACATGAATTTGAAATCGAAGGGGAAGGCGAAGGTAGACCATACGAAGGAACCCAGAC
CGCAAAGCTTAAAGTTACCAAAGGCGGGCCACTACCATTTGCATGGGATATCTTGAGCCCTCAGTTTA
TGTATGGCAGTAAGGCCTACGTTAAACACCCAGCTGATATTCCCGACTATTTGAAATTGTCTTTTCCA
GAAGGATTCAAATGGGAAAGAGTAATGAATTTCGAGGACGGCGGAGTTGTTACTGTTACTCAAGATTC
AAGTTTGCAAGACGGTGAATTTATTTACAAGGTCAAATTAAGAGGGACTAATTTCCCTAGTGATGGTC
CCGTCATGCAAAAGAAGACTATGGGTTGGGAAGCCTCATCTGAACGTATGTATCCAGAAGATGGCGCG
CTTAAGGGGGAAATTAAACAAAGATTGAAGTTAAAAGACGGTGGTCACTACGACGCGGAAGTTAAGAC
CACTTATAAAGCTAAAAAGCCCGTTCAGTTACCTGGTGCATATAACGTAAACATTAAATTGGATATCA
CTTCACATAATGAAGATTACACTATTGTGGAACAATATGAAAGAGCTGAAGGTAGGCACTCAACGGGT
GGAATGGACGAATTGTACAAATAA

A1 gRNA sequences for reporter gene targeting

CCCGCGAAATTAATACGACTCACTATAGGGAGACCACAACGGTTTCCCTCTAGAAATAATTTTGTTTA
ACTTTAAGAAGGAGATATAAATATGGCCTACGGTATCCACCGGAGACCTATGGCAGCCTCCGGCCGCC
ATAGGACACCTTTGGTTGCCAAGGGTGACCTATGGTGACCATGGGCCACCACGGGCGACCTCAGGTAT
CCTGCGGTGTCCTGCGGTTACCAAAGGCGTCCTTTGGGTTCCACCGGATACCTCCGGAC

A2 gRNA sequences for reporter gene targeting

CCCGCGAAATTAATACGACTCACTATAGGGAGACCACAACGGTTTCCCTCTAGAAATAATTTTGTTTA
ACTTTAAGAAGGAGATATAAATATGGCCTACGGTATCCACCGGAGACCTATGGCAGCCTCCGGCCGCC
ATAGGACACCTTTGGTTGCCAAGGGTGACCTATGGTGACCATGGGCCACCACGGGCGACCTCAGGTAT
CCTGCGGTGTCCTGCGGTTACCAAAGGCGTCCTTTGGGTTCCACCGGATACCTCCGGAC

A3 gRNA sequences for reporter gene targeting

CCCGCGAAATTAATACGACTCACTATAGGGAGACCACAACGGTTTCCCTCTAGAAATAATTTTGTTTA
ACTTTAAGAAGGAGATATAAATATGGCCTACGGTATCCACCGGAGACCTATGGCAGCCTCCGGCCGCC
ATAGGACACCTTTGGTTGCCAAGGGTGACCTATGGTGACCATGGGCCACCACGGGCGACCTCAGGTAT
CCTGCGGTGTCCTGCGGTTACCAAAGGCGTCCTTTGGGTTCCACCGGATACCTCCGGAC

A4 gRNA sequences for reporter gene targeting

CCCGCGAAATTAATACGACTCACTATAGGGAGACCACAACGGTTTCCCTCTAGAAATAATTTTGTTTA
ACTTTAAGAAGGAGATATAAATATGGCCTACGGTATCCACCGGAGACCTATGGCAGCCTCCGGCCGCC
ATAGGACACCTTTGGTTGCCAAGGGTGACCTATGGTGACCATGGGCCACCACGGGCGACCTCAGGTAT
CCTGCGGTGTCCTGCGGTTACCAAAGGCGTCCTTTGGGTTCCACCGGATACCTCCGGAC

A5 gRNA sequences for reporter gene targeting

CCCGCGAAATTAATACGACTCACTATAGGGAGACCACAACGGTTTCCCTCTAGAAATAATTTTGTTTA
ACTTTAAGAAGGAGATATAAATATGGCCTACGGTATCCACCGGAGACCTATGGCAGCCTCCGGCCGCC
ATAGGACACCTTTGGTTGCCAAGGGTGACCTATGGTGACCATGGGCCACCACGGGCGACCTCAGGTAT
CCTGCGGTGTCCTGCGGTTACCAAAGGCGTCCTTTGGGTTCCACCGGATACCTCCGGAC

A6 gRNA sequences for reporter gene targeting

CCCGCGAAATTAATACGACTCACTATAGGGAGACCACAACGGTTTCCCTCTAGAAATAATTTTGTTTA
ACTTTAAGAAGGAGATATAAATATGGCCTACGGTATCCACCGGAGACCTATGGCAGCCTCCGGCCGCC
ATAGGACACCTTTGGTTGCCAAGGGTGACCTATGGTGACCATGGGCCACCACGGGCGACCTCAGGTAT
CCTGCGGTGTCCTGCGGTTACCAAAGGCGTCCTTTGGGTTCCACCGGATACCTCCGGAC

A7 gRNA sequences for reporter gene targeting

CCCGCGAAATTAATACGACTCACTATAGGGAGACCACAACGGTTTCCCTCTAGAAATAATTTTGTTTA
ACTTTAAGAAGGAGATATAAATATGGCCTACGGTATCCACCGGAGACCTATGGCAGCCTCCGGCCGCC
ATAGGACACCTTTGGTTGCCAAGGGTGACCTATGGTGACCATGGGCCACCACGGGCGACCTCAGGTAT
CCTGCGGTGTCCTGCGGTTACCAAAGGCGTCCTTTGGGTTCCACCGGATACCTCCGGAC

A8 gRNA sequences for reporter gene targeting

CCCGCGAAATTAATACGACTCACTATAGGGAGACCACAACGGTTTCCCTCTAGAAATAATTTTGTTTA
ACTTTAAGAAGGAGATATAAATATGGCCTACGGTATCCACCGGAGACCTATGGCAGCCTCCGGCCGCC
ATAGGACACCTTTGGTTGCCAAGGGTGACCTATGGTGACCATGGGCCACCACGGGCGACCTCAGGTAT
CCTGCGGTGTCCTGCGGTTACCAAAGGCGTCCTTTGGGTTCCACCGGATACCTCCGGAC

A9 gRNA sequences for reporter gene targeting

CCCGCGAAATTAATACGACTCACTATAGGGAGACCACAACGGTTTCCCTCTAGAAATAATTTTGTTTA
ACTTTAAGAAGGAGATATAAATATGGCCTACGGTATCCACCGGAGACCTATGGCAGCCTCCGGCCGCC
ATAGGACACCTTTGGTTGCCAAGGGTGACCTATGGTGACCATGGGCCACCACGGGCGACCTCAGGTAT
CCTGCGGTGTCCTGCGGTTACCAAAGGCGTCCTTTGGGTTCCACCGGATACCTCCGGAC

A10 gRNA sequences for reporter gene targeting

CCCGCGAAATTAATACGACTCACTATAGGGAGACCACAACGGTTTCCCTCTAGAAATAATTTTGTTTA
ACTTTAAGAAGGAGATATAAATATGGCCTACGGTATCCACCGGAGACCTATGGCAGCCTCCGGCCGCC
ATAGGACACCTTTGGTTGCCAAGGGTGACCTATGGTGACCATGGGCCACCACGGGCGACCTCAGGTAT
CCTGCGGTGTCCTGCGGTTACCAAAGGCGTCCTTTGGGTTCCACCGGATACCTCCGGAC

A11 gRNA sequences for reporter gene targeting

CCCGCGAAATTAATACGACTCACTATAGGGAGACCACAACGGTTTCCCTCTAGAAATAATTTTGTTTA
ACTTTAAGAAGGAGATATAAATATGGCCTACGGTATCCACCGGAGACCTATGGCAGCCTCCGGCCGCC
ATAGGACACCTTTGGTTGCCAAGGGTGACCTATGGTGACCATGGGCCACCACGGGCGACCTCAGGTAT
CCTGCGGTGTCCTGCGGTTACCAAAGGCGTCCTTTGGGTTCCACCGGATACCTCCGGAC

A12 gRNA sequences for reporter gene targeting

CCCGCGAAATTAATACGACTCACTATAGGGAGACCACAACGGTTTCCCTCTAGAAATAATTTTGTTTA
ACTTTAAGAAGGAGATATAAATATGGCCTACGGTATCCACCGGAGACCTATGGCAGCCTCCGGCCGCC
ATAGGACACCTTTGGTTGCCAAGGGTGACCTATGGTGACCATGGGCCACCACGGGCGACCTCAGGTAT
CCTGCGGTGTCCTGCGGTTACCAAAGGCGTCCTTTGGGTTCCACCGGATACCTCCGGAC

A13 gRNA sequences for reporter gene targeting

CCCGCGAAATTAATACGACTCACTATAGGGAGACCACAACGGTTTCCCTCTAGAAATAATTTTGTTTA
ACTTTAAGAAGGAGATATAAATATGGCCTACGGTATCCACCGGAGACCTATGGCAGCCTCCGGCCGCC
ATAGGACACCTTTGGTTGCCAAGGGTGACCTATGGTGACCATGGGCCACCACGGGCGACCTCAGGTAT
CCTGCGGTGTCCTGCGGTTACCAAAGGCGTCCTTTGGGTTCCACCGGATACCTCCGGAC

R1 gRNA sequences for reporter gene targeting

TTGACAGCTAGCTCAGTCCTAGGGATTGTGCTAGCGAATTCATTAAAGAGGAGAAAGGTCATATGAGT
AAAGGAGAAGAACTTTTCACTGGAGTTGTCCCAATTCTTGTTGAATTAGATGGTGATGTTAATGGGCA
CAAATTTTCTGTCAGTGGAGAGGGTGAAGGTGATGCAACATACGGAAAACTTACCCTTAAATTTATTT
GCACTACTGGAAAACTACCTGTTCCATGGCCAACACTTGTCACTACTCTGACCTATGGTGTTCAATGC
TTTTCCCGTTATCCGGATCACATGAAACGGCATGACTTTTTCAAGAGTGCCATGCCCGAAGGTTATGT
ACAGGAACGCACTATATCTTTCAAAGATGACGGGAACTACAAGACGCGTGCTGAAGTCAAGTTTGAAG
GTGATACCCTTGTTAATCGTATCGAGTTAAAAGGTATTGATTTTAAAGAAGATGGAAACATTCTCGGA
CACAAACTCGAGTACAACTATAACTCACACAATGTATACATCACGGCAGACAAACAAAAGAATGGAAT
CAAAGCTAACTTCAAAATTCGCCACAACATTGAAGATGGTTCCGTTCAACTAGCAGACCATTATCAAC
AAAATACTCCAATTGGCGATGGCCCTGTCCTTTTACCAGACAACCATTACCTGTCGACACAATCTGCC
CTTTCGAAAGATCCCAACGAAAAGCGTGACCACATGGTCCTTCTTGAGTTTGTAACTGCTGCTGGGAT
TACACATGGCATGGATGAGCTCTACAAATAA

R2 gRNA sequences for reporter gene targeting

TTGACAGCTAGCTCAGTCCTAGGGATTGTGCTAGCGAATTCATTAAAGAGGAGAAAGGTCATATGAGT
AAAGGAGAAGAACTTTTCACTGGAGTTGTCCCAATTCTTGTTGAATTAGATGGTGATGTTAATGGGCA
CAAATTTTCTGTCAGTGGAGAGGGTGAAGGTGATGCAACATACGGAAAACTTACCCTTAAATTTATTT
GCACTACTGGAAAACTACCTGTTCCATGGCCAACACTTGTCACTACTCTGACCTATGGTGTTCAATGC
TTTTCCCGTTATCCGGATCACATGAAACGGCATGACTTTTTCAAGAGTGCCATGCCCGAAGGTTATGT
ACAGGAACGCACTATATCTTTCAAAGATGACGGGAACTACAAGACGCGTGCTGAAGTCAAGTTTGAAG
GTGATACCCTTGTTAATCGTATCGAGTTAAAAGGTATTGATTTTAAAGAAGATGGAAACATTCTCGGA
CACAAACTCGAGTACAACTATAACTCACACAATGTATACATCACGGCAGACAAACAAAAGAATGGAAT
CAAAGCTAACTTCAAAATTCGCCACAACATTGAAGATGGTTCCGTTCAACTAGCAGACCATTATCAAC
AAAATACTCCAATTGGCGATGGCCCTGTCCTTTTACCAGACAACCATTACCTGTCGACACAATCTGCC
CTTTCGAAAGATCCCAACGAAAAGCGTGACCACATGGTCCTTCTTGAGTTTGTAACTGCTGCTGGGAT
TACACATGGCATGGATGAGCTCTACAAATAA

R3 gRNA sequences for reporter gene targeting

TTGACAGCTAGCTCAGTCCTAGGGATTGTGCTAGCGAATTCATTAAAGAGGAGAAAGGTCATATGAGT
AAAGGAGAAGAACTTTTCACTGGAGTTGTCCCAATTCTTGTTGAATTAGATGGTGATGTTAATGGGCA
CAAATTTTCTGTCAGTGGAGAGGGTGAAGGTGATGCAACATACGGAAAACTTACCCTTAAATTTATTT
GCACTACTGGAAAACTACCTGTTCCATGGCCAACACTTGTCACTACTCTGACCTATGGTGTTCAATGC
TTTTCCCGTTATCCGGATCACATGAAACGGCATGACTTTTTCAAGAGTGCCATGCCCGAAGGTTATGT
ACAGGAACGCACTATATCTTTCAAAGATGACGGGAACTACAAGACGCGTGCTGAAGTCAAGTTTGAAG
GTGATACCCTTGTTAATCGTATCGAGTTAAAAGGTATTGATTTTAAAGAAGATGGAAACATTCTCGGA
CACAAACTCGAGTACAACTATAACTCACACAATGTATACATCACGGCAGACAAACAAAAGAATGGAAT
CAAAGCTAACTTCAAAATTCGCCACAACATTGAAGATGGTTCCGTTCAACTAGCAGACCATTATCAAC
AAAATACTCCAATTGGCGATGGCCCTGTCCTTTTACCAGACAACCATTACCTGTCGACACAATCTGCC
CTTTCGAAAGATCCCAACGAAAAGCGTGACCACATGGTCCTTCTTGAGTTTGTAACTGCTGCTGGGAT
TACACATGGCATGGATGAGCTCTACAAATAA

R4 gRNA sequences for reporter gene targeting

TTGACAGCTAGCTCAGTCCTAGGTATAATTGCTAAGCGAATTCCATTAAAGAGGAGAAAGGTACCATG
GTAAGTAAGGGTGAAGAAGACAATATGGCGATCATTAAGGAATTCATGCGTTTCAAAGTACACATGGA
GGGAAGCGTGAACGGACATGAATTTGAAATCGAAGGGGAAGGCGAAGGTAGACCATACGAAGGAACCC
AGACCGCAAAGCTTAAAGTTACCAAAGGCGGGCCACTACCATTTGCATGGGATATCTTGAGCCCTCAG
TTTATGTATGGCAGTAAGGCCTACGTTAAACACCCAGCTGATATTCCCGACTATTTGAAATTGTCTTT
TCCAGAAGGATTCAAATGGGAAAGAGTAATGAATTTCGAGGACGGCGGAGTTGTTACTGTTACTCAAG
ATTCAAGTTTGCAAGACGGTGAATTTATTTACAAGGTCAAATTAAGAGGGACTAATTTCCCTAGTGAT
GGTCCCGTCATGCAAAAGAAGACTATGGGTTGGGAAGCCTCATCTGAACGTATGTATCCAGAAGATGG
CGCGCTTAAGGGGGAAATTAAACAAAGATTGAAGTTAAAAGACGGTGGTCACTACGACGCGGAAGTTA
AGACCACTTATAAAGCTAAAAAGCCCGTTCAGTTACCTGGTGCATATAACGTAAACATTAAATTGGAT
ATCACTTCACATAATGAAGATTACACTATTGTGGAACAATATGAAAGAGCTGAAGGTAGGCACTCAAC
GGGTGGAATGGACGAATTGTACAAATAA

Annotations: Upstream region, BBa_J23117 promoter, BBa_J23119 promoter, RBS, GFP, mCherry, gRNA targeting the template strand, gRNA targeting the non-template strand

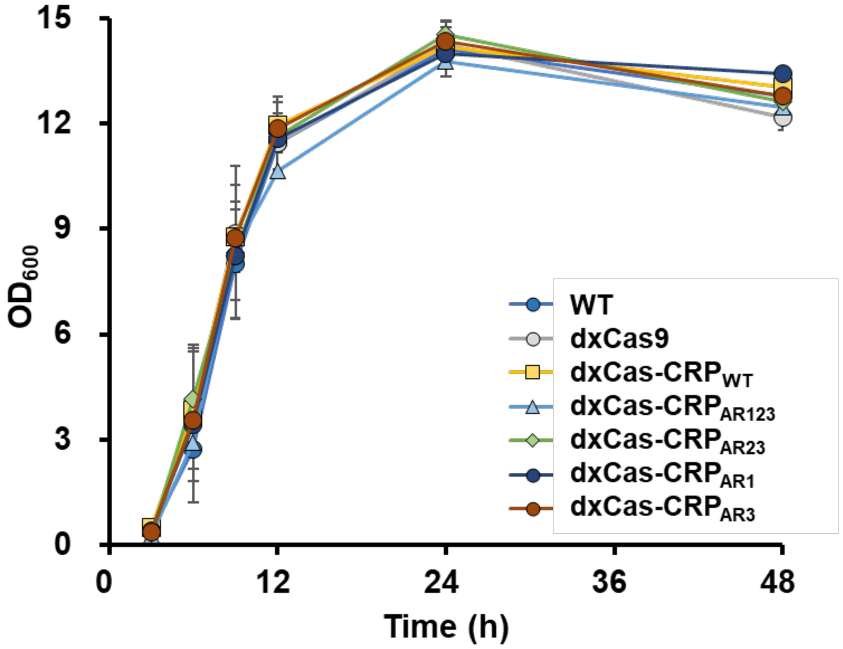

Supplementary Figure S2. Growth curves of *E. coli* strains expressing dxCas9 or dxCas9–CRP derivatives compared to wild-type (WT) *E. coli*. Cells were cultured in LB medium at 37 °C with shaking at 200 rpm for 48 h. Expression of dxCas9 was induced with 1 mM L-rhamnose. Data represent the mean of biological triplicates; error bars indicate standard deviation.

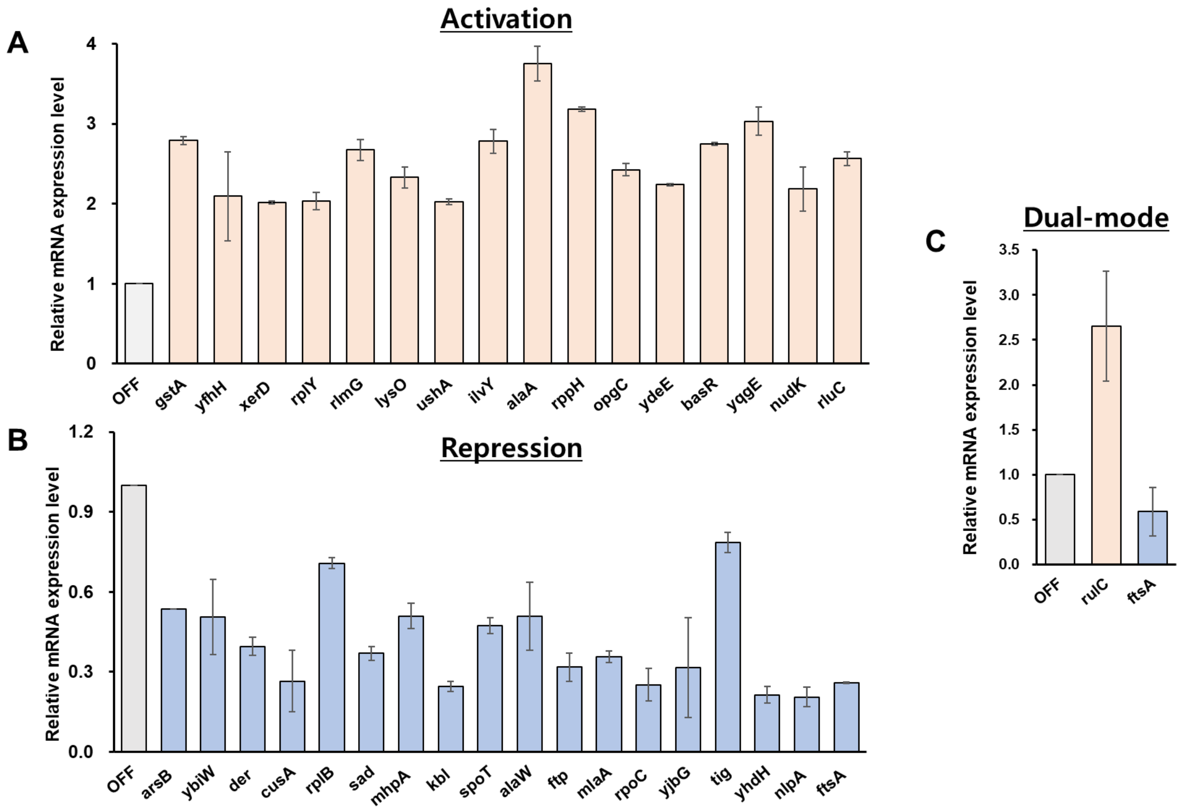

Supplementary Figure S3. Transcriptional changes of CRISPRa/i-targeted genes in violacein-producing *E. coli* strains. mRNA levels were measured by qRT-PCR in selected strains from the genome-wide screen. **(A)** All screened genes showed increased expression compared to the off-target control strain (gray), confirming successful transcriptional activation. **(B)** All targets selected from the CRISPRi screen exhibited reduced expression relative to the off-target control strain (gray), validating the efficacy of CRISPRi-mediated repression. **(C)** Transcriptional profile of a strain co-expressing gRNAs for *rluC* activation and *ftsA* repression. The simultaneous upregulation of *rluC* and downregulation of *ftsA* supports the synergistic increase in violacein production. qRT-PCR analysis was performed using violacein-producing strains harvested during the exponential growth phase (at 8 h). Expression values are shown as fold changes normalized to the off-target control strain. Bars indicate mean ± standard deviation from three independent biological replicates.

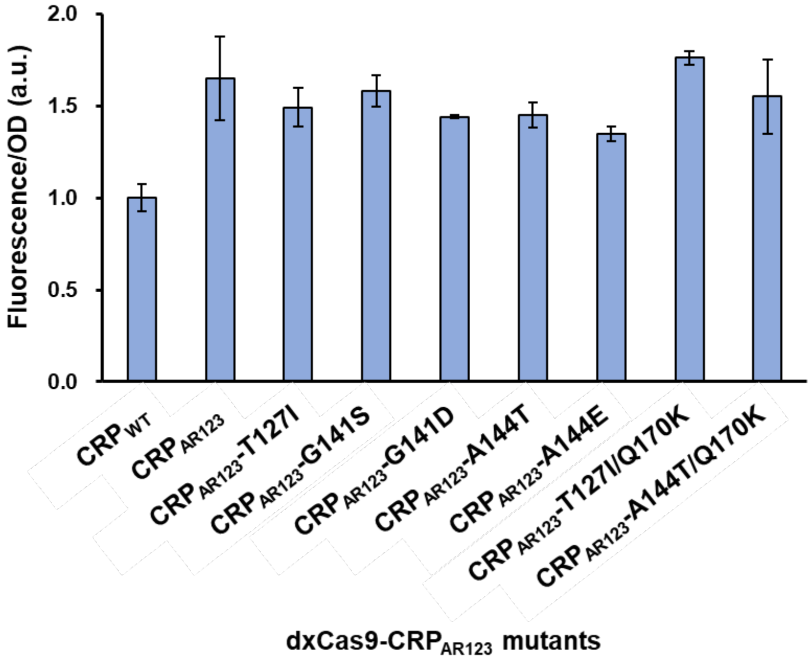

Supplementary Figure S4. CRISPRa driven by dxCas9 combined with cAMP-independent CRP mutants (dxCas9-CRP$_{mutant}$). The cAMP-independent CRP mutants included T127I, G141S, G141D, A144T, A144E, T127I/Q170K, and A144T/Q170K, each lacking the native DNA-binding domain. The graph shows the relative fluorescence intensities of the GFP reporter driven by dxCas9-CRP$_{mutant}$ and an activating gRNA (A7, GFP-gRNAa targeting at -191 bp). All cAMP-independent CRP mutants showed comparable activation to the CRP derivative (CRP$_{AR123}$). All data shown are from at least three biological replicates, and the error bars indicate the standard deviation.

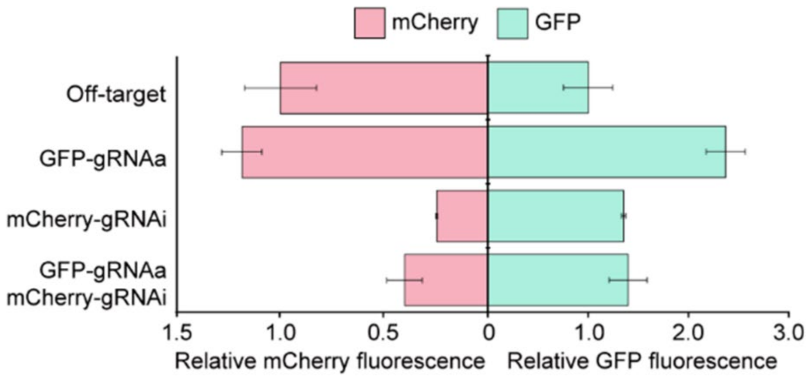

Supplementary Figure S5. Simultaneous activation and repression of multiple genes using the CRISPRa/i system in *P. putida*. The cross-species functionality of the dxCas9–CRP_{AR123} system was evaluated using a dual-fluorescent reporter system with their corresponding gRNAs in *P. putida* KT2440. The graph shows the relative fluorescence intensities of GFP and mCherry in response to individual or combined gRNAs targeting GFP for activation (GFP-gRNAa) and mCherry for repression (mCherry-gRNAi). When both gRNAs were applied simultaneously, we observed concurrent GFP activation (1.40-fold) and mCherry repression (0.40-fold), demonstrating the dual-mode regulatory capability of the dxCas9–CRP_{AR123} system. All data represent the mean of at least three biological replicates, and error bars indicate standard deviation.

Supplementary Note 1. CRP as the effector domain for CRISPRa

CRP (cyclic AMP receptor protein) is a well-characterized global transcriptional regulator in *E. coli*, known to control the expression of over 400 genes involved in carbon metabolism, nutrient uptake, and energy homeostasis [7, 8]. Mechanistically, CRP binds to specific DNA sequences located near target promoters and activates transcription by facilitating the recruitment of RNA polymerase through direct protein–protein interactions [9-11]. Its activating regions—AR1 and AR2—interact with the C-terminal domain of the RNA polymerase α-subunit, while AR3 contacts the σ^{70} subunit. These multi-site interactions enhance the binding of RNA polymerase to promoters and promote efficient promoter clearance, making CRP a potent transcriptional activator.

In this study, we selected CRP as the effector domain for CRISPRa due to the several advantageous features. First, CRP is endogenous to *E. coli*, ensuring compatibility with the native transcriptional machinery, and it activates a broad set of σ^{70}-dependent promoters, which regulate the majority of gene expression in *E. coli* [12,13]. Additionally, the well-characterized structure-function relationships of CRP allow for rational, modular engineering of its activation regions. Its relatively small size also enables direct fusion to dxCas9 without necessitating complex multi-component systems. Furthermore, CRP is highly conserved across diverse bacterial species, supporting its potential as a versatile and broadly applicable effector domain for bacterial CRISPRa systems [12,13].

Regarding cAMP dependency, in *E. coli*, CRP is activated through binding to cAMP, which induces a conformational change required for specific DNA binding. Specifically, in the absence of cAMP, apo-CRP adopts an "off" state that binds DNA nonspecifically and weakly [10,14]. Upon cAMP binding to its N-terminal domain, CRP undergoes an allosteric transition to the "on" state, which binds DNA specifically and strongly via its C-terminal domain [14]. However, in our system, we used CRP derivatives lacking the native DNA-binding domain and instead relied on dxCas9 for DNA targeting. Because our system does not depend on the native DNA-binding domain of CRP and the effector domain is artificially recruited to target DNA by dxCas9, cAMP binding is not essential in this study. Rather, CRP derivatives are expected to function solely as activation domains, acting independently of their native DNA-binding activity.

To further investigate the role of cAMP dependency in our CRISPRa system utilizing CRP derivatives, we tested several previously characterized cAMP-independent CRP mutants—including T127I, G141S, G141D, A144T, A144E, T127I/Q170K, and A144T/Q170K—fused to dxCas9 [15]. Notably, these CRP mutants, which also lack the native DNA-binding domain, exhibited transcriptional activation levels comparable to those of our CRP derivative (CRP_{AR123}) (**Supplementary Fig. S4**). These results indicate that cAMP-mediated allosteric activation is not required in our CRISPRa design, provided that the CRP effector is artificially recruited to target loci via dxCas9.

Supplementary Note 2. Compatibility of the dxCas9-CRP System in *P. putida* KT2440.

To evaluate the cross-species compatibility, we applied our system in *P. putida* KT2440. The dxCas9-CRP_{AR123} construct was placed under P_{Lac} promoter instead of ${}_{rha}P_{BAD}$, as the rhamnose-inducible system is non-functional in *P. putida*. In addition, the BBR1 origin of replication was used for stable plasmid maintenance in *P. putida*. The dual-fluorescent reporter system and gRNAs were cloned into a single plasmid along with the dxCas9-CRP_{AR123} system (**Supplementary Table S2**). Cultures were grown at 30 °C and 0.1 mM IPTG was used for induction. All other culture and analysis procedures were conducted similarly to the *E. coli* described in the main manuscript.

Our dxCas9-CRP_{AR123} system successfully demonstrated dual-mode regulation in *P. putida* KT2440 (**Supplementary Fig. S5**). The system achieved 2.37-fold activation of GFP expression for gene activation (gRNA_A7) and substantial repression of mCherry expression (0.24-fold) for gene repression (gRNA_R4), when targeting individual genes. When both activation and repression were applied simultaneously, the system maintained dual-mode capability with concurrent 1.40-fold GFP activation and 0.40-fold mCherry expression, demonstrating its ability to regulate multiple genes simultaneously.

Despite the moderate sequence identity (~63%) between *E. coli* and *P. putida* CRP (PP_0424) proteins [16], the successful demonstration suggests that our *E. coli* CRP-based system retains cross-species compatibility and indicates promising potential for broader applications. CRP orthologs are widely distributed across diverse bacterial species [12]. While overall performance was slightly reduced compared to *E. coli*, potentially due to species-specific differences in transcriptional machinery in *P. putida*, these limitations could be overcome through further optimization or the utilization of species-specific CRP domain variants. These results suggest the broad applicability of our system across phylogenetically distinct bacterial species and serve as a universal bacterial regulation platform.

Supplementary Materials and Methods. RT-qPCR analysis.

E. coli cultures were grown for 8 h to an OD_{600} of approximately 4.0, harvested by centrifugation, and total RNA was extracted using the RNeasy Mini Kit (Qiagen, Hilden, Germany) according to the manufacturer's protocol, including on-column DNase I treatment to remove residual genomic DNA. For each sample, 100 ng of total RNA was reverse-transcribed into cDNA using the SuperScript III First-Strand Synthesis System (Life Technologies, OR, USA) with random hexamer primers in a final reaction volume of 20 µL.

qPCR reactions were performed in technical triplicate using iTaq Universal SYBR Green Supermix (Bio-Rad Laboratories, Hemel Hempstead, UK) on a CFX96™ Real-Time PCR Detection System (Bio-Rad). Gene-specific primers are listed below. The thermal cycling conditions were as follows: 95 °C for 30 s, followed by 40 cycles of 95 °C for 5 s and 60 °C for 5 s, with a melt-curve analysis from 65 to 95 °C in 0.5 °C increments. Relative transcript levels were calculated using the $2^{-\Delta\Delta Ct}$ method, with *E. coli* mRNA levels normalized to the 16S rRNA gene. Three independent biological replicates were analyzed for each condition.

Primers used for RT-qPCR analysis

Target	Primer name	Sequence	PCR product size (bp)
Activation			
lysO	lysO_F	TCTTACAACACGCGACCGAA	137
	lysO_R	CACCACGGCGACAATCATTC	
opgC	opgC_F	TCAGCGCGGGTGACTTATTT	105
	opgC_R	GGAGGTGATGTGCGGTGTAA	
basR	basR_F	GTACTGATCCTCACCGCTCG	138
	basR_R	ATTATGGCGTCGTAGCAGGG	
xerD	xerD_F	CTGCGTGTCTCTGAACTGGT	128
	xerD_R	AGCCAGTAAACCGCCTCTTC	
rplY	rplY_F	TTCCCGGCAATCATCTACGG	115
	rplY_R	CAGCGAAGTT CTGACCATCG	
rlmG	rlmG_F	CGCCGTTTCACCAACAACAT	121
	rlmG_R	TAATCCAGGTGACGGTTGGC	
ilvY	ilvY_F	CGGCAGATGCGATGGAAAAG	109
	ilvY_R	GCCAGATTCTCCAGCATCGA	
rppH	rppH_F	GCGGCGATGCAGAAATCAAT	148
	rppH_R	ACCACACTCGCGAACTCTTT	
rluC	rluC_F	AGAGCGTTCAAGCACCGTTA	130
	rluC_R	ACCAGGGTGGCAAATGCATA	
gstA	gstA_F	TCTGTTTACGGTTCTGCGCT	118
	gstA_R	TGACAGCGCGTCTTGTACTT	
ushA	ushA_F	GCTGATTCCGGTGAACCTGA	139
	ushA_R	CTGCGCTTTGCCTTTGTTCT	
alaA	alaA_F	CTTTACTCCGCGCGTAAAGC	113

	alaA_R	GCCTGAACGATAAGCTCCGA	
nudK	nudK_F	CGCTACCTGGGTTAATGGCA	141
	nudK_R	AATTTGCGCACTTCACCCAC	
yqgE	yqgE_F	CGCCCTCCAATTTTGCTTCC	123
	yqgE_R	AACCCAGAGCCACCAATACG	
yfhH	yfhH_F	AGCCATTTCCTACACCGGTG	105
	yfhH_R	AAAGGTTTTC CGGCGCTTAA	
ydeE	ydeE_F	ACTTGAGTCGCCAGTACAGC	106
	ydeE_R	TCCGCCAGGATACCAAAACC	
Repression			
ftsA	ftsA_F	CTCAGCGCGTGTTTCATACG	100
	ftsA_R	CCCACCGCCGTCGAATAATA	
nlpA	nlpA_F	TCGCGCACTTTTACTGTTGC	156
	nlpA_R	TTGGGATCATCCAGTACGCG	
cusA	cusA_F	ATCCCGATCTTCACCCTGGA	108
	cusA_R	GATCACTACGATCGCCAGCA	
der	der_F	TGATACCGGCGGGATTGATG	111
	der_R	CGCGCATCCACCATAAACAG	
rplB	rplB_F	TTCAGTCTGGCGTTGATGCT	113
	rplB_R	CTGACCGCCTTTACCTGGTT	
spoT	spoT_F	TGCCCATTAAAGGTGCCGAT	141
	spoT_R	AGCCACGGATATTACGGCAG	
alaW	alaW_F	GGGGCTATAGCTCAGCTGGGAG	80
	alaW_R	GTGCAGGGTCCGAGGT	
rpoC	rpoC_F	GATTCCGAAATGGCGTCAGC	124
	rpoC_R	GTGGTGTTCATGCTGTTACT	
tig	tig_F	ACCATCGACTTCACCGGTTC	108
	tig_R	GTCTTCAAAGCCCGGGATCA	
sad	sad_F	ACCATCGACTTCACCGGTTC	108
	sad_R	GTCTTCAAAGCCCGGGATCA	
mhpA	mhpA_F	GCAAAGTGCTGCCTAATCCG	130
	mhpA_R	CAACGCGCGACTGGCGCAAC	
kbl	kbl_F	TTTGATGCTAACGGTGGCCT	157
	kbl_R	GTGCTTCCAGCTCCTGCATA	
yhdH	yhdH_F	GCAAAGTGCTGCCTAATCCG	118
	yhdH_R	CTGCTGGCGGGCGATGCCGC	
ftp	ftp_F	ATGGCCGATATCGTCACCAC	163
	ftp_R	CGGTTTTGGCTTTCATCGCA	
arsB	arsB_F	CTACGATATGGCGCTGCTGA	164
	arsB_R	ATAATATCAGCGCGCCCACA	
mlaA	mlaA_F	TAGAAGGGTTCAACCGCACC	106
	mlaA_R	GCCGGTTGCGGAACATAATC	
ybiW	ybiW_F	ATGCTGGAAGAGAACGTGCA	114
	ybiW_R	CAGGCCAGAAACCCAGTCAT	
yjbG	yjbG_F	TCGCGTTGCTTTTGAGTGTG	110
	yjbG_R	TAAATGCTCTGCGCCCGTTA	

Reference

[1] Fontana, J., Dong, C., Kiattisewee, C., Chavali, V.P., Tickman, B.I., Carothers, J.M. and Zalatan, J.G. (2020) Effective CRISPRa-mediated control of gene expression in bacteria must overcome strict target site requirements. *Nat Commun*, **11**, 1618.

[2] Liu, Y., Wan, X. and Wang, B. (2019) Engineered CRISPRa enables programmable eukaryote-like gene activation in bacteria. *Nat Commun*, **10**, 3693.

[3] Dong, C., Fontana, J., Patel, A., Carothers, J.M. and Zalatan, J.G. (2018) Synthetic CRISPR-Cas gene activators for transcriptional reprogramming in bacteria. *Nat Commun*, **9**, 2489.

[4] Bikard, D., Jiang, W., Samai, P., Hochschild, A., Zhang, F. and Marraffini, L.A. (2013) Programmable repression and activation of bacterial gene expression using an engineered CRISPR-Cas system. *Nucleic Acids Res*, **41**, 7429-7437.

[5] Kim, S. K., Han, G. H., Seong, W., Kim, H., Kim, S. W., Lee, D. H., & Lee, S. G. (2016). CRISPR interference-guided balancing of a biosynthetic mevalonate pathway increases terpenoid production. *Metabolic engineering*, *38*, 228-240.

[6] Kwon, S. K., Kim, S. K., Lee, D. H., Kim, J. (2015). Comparative genomics and experimental evolution of Escherichia coli BL21(DE3) strains reveal the landscape of toxicity escape from membrane protein overproduction. *Scientific Reports*, *5*, 16076

[7] Shimada, T., Fujita, N., Yamamoto, K. and Ishihama, A. (2011) Novel roles of cAMP receptor protein (CRP) in regulation of transport and metabolism of carbon sources. *PLoS One*, **6**, e20081.

[8] Gancedo, J.M. (2013) Biological roles of cAMP: variations on a theme in the different kingdoms of life. *Biol Rev Camb Philos Soc*, **88**, 645-668.

[9] Sondberg, E., Sinha, A.K., Gerdes, K. and Semsey, S. (2019) CRP Interacts Specifically With Sxy to Activate Transcription in Escherichia coli. *Front Microbiol*, **10**, 2053.

[10] Lawson, C.L., Swigon, D., Murakami, K.S., Darst, S.A., Berman, H.M. and Ebright, R.H. (2004) Catabolite activator protein: DNA binding and transcription activation. *Curr Opin Struct Biol*, **14**, 10-20.

[11] Busby, S. and Ebright, R.H. (1999) Transcription activation by catabolite activator protein (CAP). *J Mol Biol*, **293**, 199-213.

[12] Soberon-Chavez, G., Alcaraz, L.D., Morales, E., Ponce-Soto, G.Y. and Servin-Gonzalez, L. (2017) The Transcriptional Regulators of the CRP Family Regulate Different Essential Bacterial Functions and Can Be Inherited Vertically and Horizontally. *Front Microbiol*, **8**, 959.

[13] Krol, E., Werel, L., Essen, L.O. and Becker, A. (2023) Structural and functional diversity of bacterial cyclic nucleotide perception by CRP proteins. *Microlife*, **4**, uqad024.

[14] Benoff, B., Yang, H., Lawson, C.L., Parkinson, G., Liu, J., Blatter, E., Ebright, Y.W.,

Berman, H.M. and Ebright, R.H. (2002) Structural basis of transcription activation: the CAP-αCTD-DNA complex. *Science*, **297**, 1562-1566.

[15] Frendorf, P.O., Lauritsen, I., Sekowska, A., Danchin, A. and Norholm, M.H.H. (2019) Mutations in the Global Transcription Factor CRP/CAP: Insights from Experimental Evolution and Deep Sequencing. *Comput Struct Biotechnol J*, **17**, 730-736.

[16] Milanesio, P., Arce-Rodriguez, A., Munoz, A., Calles, B. and de Lorenzo, V. (2011) Regulatory exaptation of the catabolite repression protein (Crp)-cAMP system in Pseudomonas putida. *Environ Microbiol*, **13**, 324-339.
